# CERENA: ChEmical REaction Network Analyzer—A Toolbox for the Simulation and Analysis of Stochastic Chemical Kinetics

**DOI:** 10.1371/journal.pone.0146732

**Published:** 2016-01-25

**Authors:** Atefeh Kazeroonian, Fabian Fröhlich, Andreas Raue, Fabian J. Theis, Jan Hasenauer

**Affiliations:** 1 Institute of Computational Biology, Helmholtz Zentrum München—German Research Center for Environmental Health, Neuherberg, Germany; 2 Department of Mathematics, Chair of Mathematical Modeling of Biological Systems, Technische Universität München, Garching, Germany; 3 Merrimack Pharmaceuticals Inc., Discovery Devision, Cambridge, MA 02139, United States of America; University of Calgary, CANADA

## Abstract

Gene expression, signal transduction and many other cellular processes are subject to stochastic fluctuations. The analysis of these stochastic chemical kinetics is important for understanding cell-to-cell variability and its functional implications, but it is also challenging. A multitude of exact and approximate descriptions of stochastic chemical kinetics have been developed, however, tools to automatically generate the descriptions and compare their accuracy and computational efficiency are missing. In this manuscript we introduced CERENA, a toolbox for the analysis of stochastic chemical kinetics using Approximations of the Chemical Master Equation solution statistics. CERENA implements stochastic simulation algorithms and the finite state projection for microscopic descriptions of processes, the system size expansion and moment equations for meso- and macroscopic descriptions, as well as the novel conditional moment equations for a hybrid description. This unique collection of descriptions in a single toolbox facilitates the selection of appropriate modeling approaches. Unlike other software packages, the implementation of CERENA is completely general and allows, e.g., for time-dependent propensities and non-mass action kinetics. By providing SBML import, symbolic model generation and simulation using MEX-files, CERENA is user-friendly and computationally efficient. The availability of forward and adjoint sensitivity analyses allows for further studies such as parameter estimation and uncertainty analysis. The MATLAB code implementing CERENA is freely available from http://cerenadevelopers.github.io/CERENA/.

## Introduction

Biological processes, including chemical reaction networks, are dynamical systems with inherently stochastic dynamics due to the discrete nature of matter [1]. The kinetics of these processes are described by continuous-time Markov chains and can be simulated using stochastic simulation algorithms (SSAs) [2]. The impact of stochastic fluctuations is more pronounced in low copy-number regimes [3] and tends to decrease, but possibly remaining important, as copy-numbers increase [4]. Given the importance of stochasticity in the dynamics of biological systems, e.g., cellular mechanisms and their functions [5], a holistic understanding of cell biology requires accurate capturing of stochastic effects.

Using well-mixed and thermal equilibrium assumptions, the dynamics of chemical reaction networks is exactly described by the Chemical Master Equation (CME) [6]. The solution of the CME yields the probability distribution over the state of the system [1]. Besides special cases [7], the exact solution of the CME is mostly infeasible as CMEs are usually infinite-dimensional systems of differential equations. Several approaches have been developed to approximate the solution of the CME, amongst others variants of finite state projection (FSP) [8–10]. However, high computational complexity is a limiting factor for the applicability of this class of simulation methods.

To reduce computational complexity, a multitude of approaches have been introduced that, instead of approximating the full probability distribution, focus on the statistical moments of it. Various orders of the method of moments (MM) [11] and the system size expansion (SSE) [1, 12] provide information about the mean and higher-order moments of the distribution. These methods yield the reaction rate equations (RRE) as a special case. To improve upon the approximation in the presence of low as well as high copy-number species, hybrid microscopic-mesoscopic approaches such as the method of conditional moments (MCM) [13] and the conditional linear noise approximations [14] have been introduced. All these methods are of reduced computational complexity as they possess significantly fewer state-variables compared to the CME or FSP, thus remain feasible for real-world application problems.

Beyond fast numerical simulation, moment-based descriptions facilitate parameter estimation and model selection for stochastic processes [15, 16]. This is essential for inferring unknown rate constants and pathway topologies from experimental data. In addition to the approximative model, state-of-the-art estimation algorithms strongly benefit from the solution of sensitivity equations [17].

**Fig 1:**
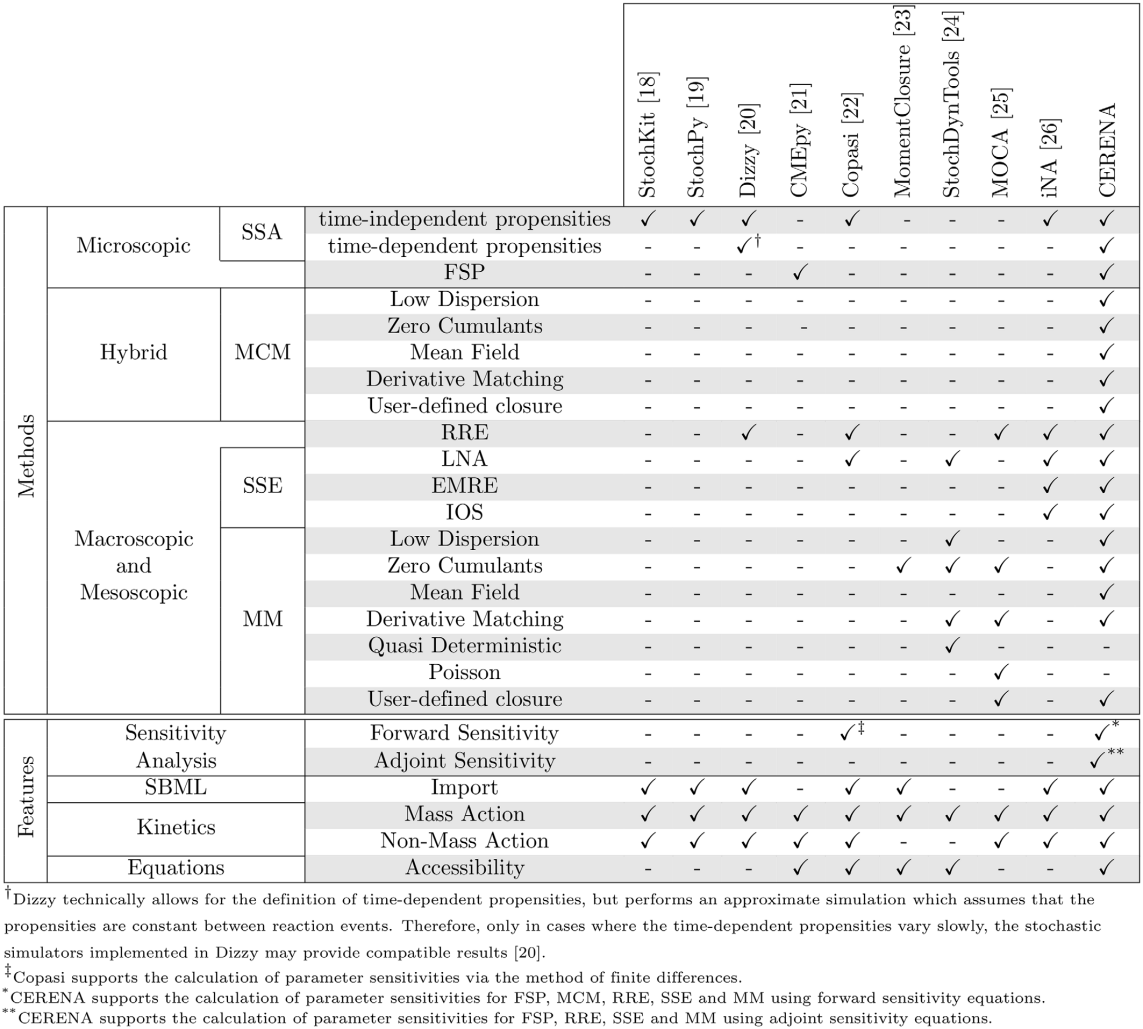
Overview of software packages for stochastic modeling and their capabilities.

Several well-known open-source software packages are available for stochastic simulations, finite state projection, method of moments, and system size expansion (e.g., [18–26] whose properties are summarized in Fig 1). In addition, there exist web-based simulation platforms, e.g., SHAVE [27]. However, a software package offering a broad collection of simulation methods is still missing. Furthermore, none of the available software provides sensitivity equations, or hybrid approaches such as the method of conditional moments.

In this paper, we introduce CERENA (ChEmical REaction Network Analyzer), a toolbox for the analysis of stochastic chemical kinetics. CERENA includes a variety of methods for the analysis of stochastic biochemical reaction networks, focusing on mesoscopic and macroscopic descriptions, namely RRE, MM, and SSE. Also, CERENA provides the first implementation of MCM, and offers a wide range of options, amongst others variable truncation orders and different closure schemes. In addition, FSP and SSA implemented in CERENA can be used to provide microscopic descriptions of stochastic chemical kinetics. Although efficient implementations of many variants of SSA are available, e.g., in StochKit [18], CERENA is the only package supporting arbitrary, including fast-varying, time-dependent reaction propensities [28]. This variety of descriptions renders CERENA unique compared to other relevant software packages (see Fig 1). To improve applicability of CERENA for realistic systems, the toolbox allows for multiple compartments, non-mass action kinetics, and time-dependent propensities. CERENA is the first toolbox for stochastic modeling to provide forward and adjoint sensitivity equations to facilitate efficient parameter estimation when linked to optimization packages. Ensuring efficient numerical simulation, CERENA enables comprehensive studies for a variety of meso- and macroscopic descriptions.

In the following, we describe the functionality of CERENA and introduce the different approximations. CERENA is then used for a detailed quantitive comparison of different approximation methods, including various moment closures, which was not done before. In particular, the approximation accuracies and computation times are assessed, demonstrating the efficiency of the CERENA implementation.

## Methods

In the following, several methods for the modeling of stochastic processes and the corresponding sensitivity analysis are briefly introduced.

### Modeling approaches for stochastic biochemical reaction networks

A chemical reaction network, comprising of *n*_*s*_ chemical species and *n*_*r*_ chemical reactions is described using a continuous-time Markov chain (CTMC) [29]. The state vector of this CTMC, $X\in {\mathbb{N}}_{0}^{n_{s}}$, represents the counts of species, and is changed every time a reaction fires. The probability of observing the CTMC at a particular state **x** at time *t* is denoted by *p*(**x**|*t*). The time evolution of the probability distribution *p*(**x**|*t*) is governed by the CME, which is a system of ordinary differential equations (ODEs) (see S1 CERENA Documentation for more details). As solving the CME is mostly infeasible due to the large or infinite number of states **x**, various approximative methods have been developed. Several methods concentrate on the full distribution *p*(**x**|*t*) to provide a microscopic description. For mesoscopic and macroscopic descriptions, there exist several methods that focus on representing the solution of the CME in terms of its statistical moments. The microscopic, mesoscopic and hybrid methods implemented in CERENA are briefly introduced in the following.

#### Stochastic Simulation Algorithm

SSAs generate statistically representative sample paths of the CTMC [2]. An estimate to the probability distribution *p*(**x**|*t*) is given by the frequency of sample paths that occupy state **x** at time *t*. To estimate the moments of the process, Monte-Carlo integration can be performed. While estimators for probability distribution and moments are unbiased and converge, the sample-sizes required to obtain low-variance estimates are generally large, rendering SSA-based methods computationally demanding.

#### Finite State Projection

To enable a direct approximation of *p*(**x**|*t*), FSP [8] reduces the number of state variables of the CME by only considering the states of non-negligible probabilities. The remaining set of ODEs then yields a lower bound for *p*(**x**|*t*). Growing the state-space of FSP decreases the approximation error at the cost of increased computational complexity.

#### Reaction Rate Equations

The RRE is the most commonly used modeling approach for biochemical reaction networks. It constitutes a system of ODEs for the time evolution of the mean of the stochastic process in the macroscopic limit. For reaction networks with constant and linear propensities, i.e. those with only zero- or monomolecular reactions, the solution of the RRE is exactly the mean of the stochastic process. For reaction networks with nonlinear propensities, the RRE prediction can be considerably different from the true mean of the process since it neglects the stochastic effects. In such cases, the solution of RRE is reflective of the true mean of the stochastic process only in the limit of large molecule numbers [30].

#### System Size Expansion

For a systematic approximation of the dynamics of mesoscopic systems, the SSE has been introduced [1]. The SSE is a power series expansion of the CME in the inverse volume of the system. The lowest-order approximation for the mean reproduces the aforementioned RRE. For the covariance, the lowest-order approximation yields the well-known linear noise approximation (LNA), whose validity has been studied in [30] for different classes of reaction systems. Higher-order corrections for the mean and covariance yield the effective mesoscopic rate equation (EMRE) [12] and the inverse omega square (IOS) approximation [26].

#### Method of Moments

The method of moments (MM) [11] is conceptually similar to SSE in that it also sets a framework for describing the moments of the solution of the CME. A system of ODEs for the exact time evolution of the moments, which constitutes the moment equations, can be derived from the CME. Generally, the equations for the lower-order moments depend on the higher-order moments, rendering moment closure necessary. Commonly used closure techniques include low dispersion closure, mean field closure, zero cumulants closure, and derivative matching closure [31]. The application of moment closure yields a closed set of approximative equations for the time evolution of the moments.

#### Method of Conditional Moments

The MCM [13] combines a microscopic description of low copy-number species with a moment-based description of high copy-number species, providing a hybrid approach for approximating the solution of the CME. Since stochastic fluctuations are more dominant for low copy-number species, marginal probability densities for these species are determined. The high-copy number species are merely described in terms of their moments, conditioned on the state of low-copy number species. The MCM equations are derived from the CME, and form a system of differential algebraic equations (DAEs). Similar to the moment equations, the moment closure is generally required to close the set of MCM equations. This hybrid description can yield an improved approximation accuracy [13].

### Sensitivity analysis

FSP, RRE, SSE, MM and MCM yield systems of differential equations. The parameters of differential equations can efficiently be inferred using gradient-based optimization methods [17]. While gradients can be approximated using finite differences, methods based on sensitivity equations are known to be more robust and computationally more efficient [17]. CERENA enables first- and second-order forward sensitivity analysis for all ODE-based and DAE-based modeling approaches, as well as adjoint sensitivity analysis [32] for all ODE-based modeling approaches.

#### Forward sensitivity equations

Forward sensitivity equations provide the time-dependent sensitivity of the state-variables of the differential equations with respect to the parameters. Assuming that the model possesses *n* state-variables and *n*_*θ*_ parameters, roughly a system of *n*(1+*n*_*θ*_) differential equations is solved to compute the first-order state sensitivities with respect to all parameters. The sensitivity of measured quantities and objective functions can then be computed based on state sensitivities.

#### Adjoint sensitivity equations

If the sensitivity of few functions with respect to many parameters is required, computing the state sensitivities is unnecessarily demanding. In this case, the adjoint sensitivity equations [32] can be solved to yield a set of adjoint states which are independent of the parameters. These trajectories are then used to calculate the sensitivity with respect to any parameters of interest, with low computational cost. Thus, in applications with high-dimensional parameter spaces and/or few output functionals, calculating adjoint sensitivities tends to be computationally more advantageous. In parameter estimation, the likelihood function can be defined as the sole output functional of the system.

## Implementation

**Fig 2:**
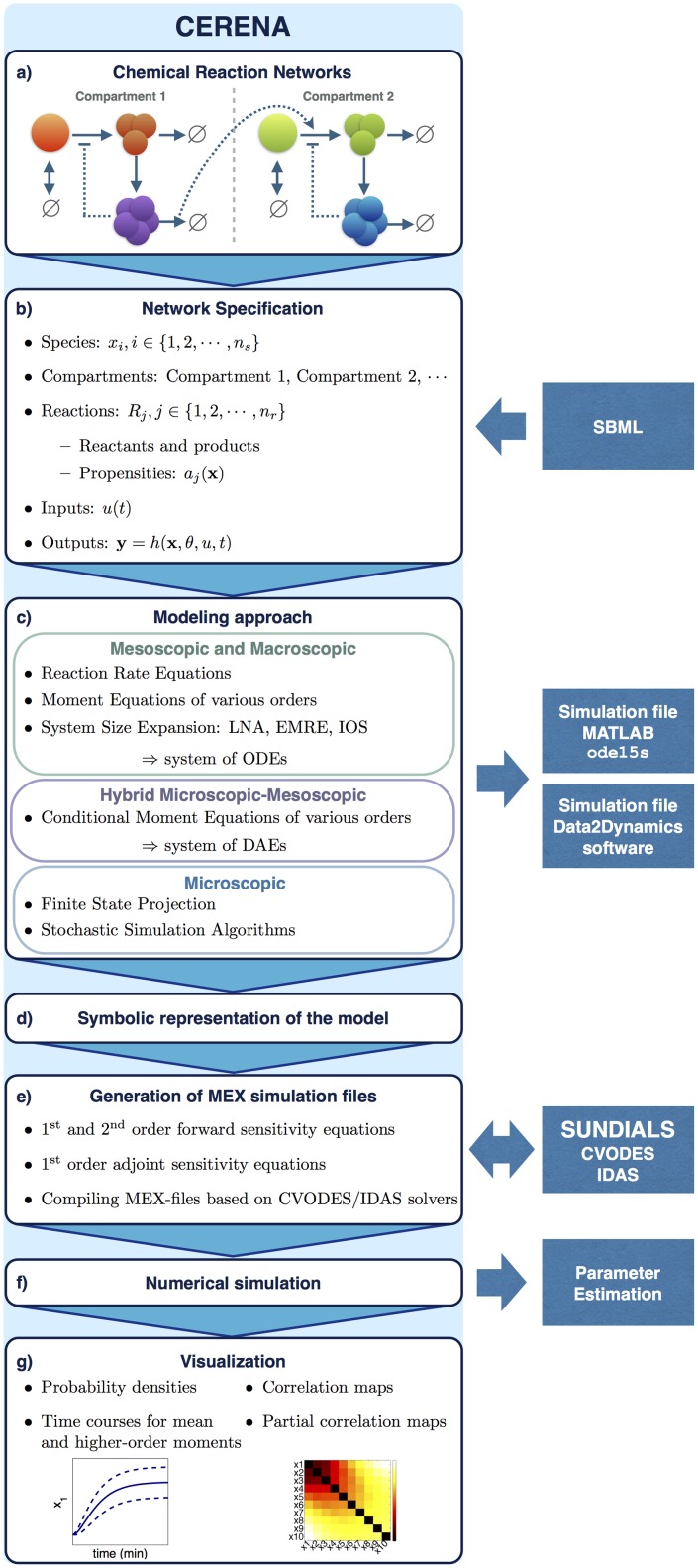
Workflow of CERENA. (a) CERENA can be used to study (multi-compartment) chemical reaction networks. (b) The reaction network can be defined in MATLAB, or alternatively, imported from SBML. (c) The system of equations for different modeling approaches implemented in CERENA is generated, and optionally stored as MATLAB functions for numerical simulation using MATLAB ODE solvers. Furthermore, the representation of the system can be exported to the estimation toolbox Data2Dynamics. (d) The symbolic representation of the system of equations together with the initial conditions is stored in a MATLAB script. (e) Based on the symbolic representation, 1^st^ and 2^nd^ order sensitivity equations are derived. MEX-files, which use CVODES and IDAS packages of SUNDIALS for the numerical simulation of the models, are compiled. (f) The generated MEX-files are used for numerical simulation, and can be integrated with other software for parameter estimation. (g) Various aspects of the simulation results can be visualized using CERENA.

CERENA is a MATLAB-based toolbox for the simulation of chemical reaction networks. It provides a collection of methods for the analysis of stochastic processes, focusing on SSE, MM and MCM of various orders. In addition, FSP and SSAs are implemented in CERENA to provide microscopic descriptions of the process, and can also be used to assess the approximation errors of the aforementioned methods. The workflow of the toolbox is laid out in Fig 2. In the following, different aspects of implementation and features of the toolbox are explained. For a detailed list of functions, we refer to the S1 CERENA Documentation. The CERENA toolbox is freely available from http://cerenadevelopers.github.io/CERENA/.

### Network specification

To use CERENA, the biochemical reaction network has to be defined in a specific format described in the S1 CERENA Documentation. The definition includes species, compartments, reactions and their propensities, inputs and observables of the system. Reaction propensities can be time-dependent and may or may not follow the law of mass action. In case of non-mass action kinetics the propensities are approximated, e.g., using Taylor series expansion in MM and MCM [33]. Inputs are used to describe experimental conditions. Alternatively, networks described in the Systems Biology Markup Language (SBML) can be imported.

### Model derivation and symbolic representation

Following the definition of the biochemical reaction network, a modeling approach and corresponding options, such as approximation order and moment closure technique, can be selected. In addition to the moment closure techniques implemented in CERENA (see Fig 1), user-defined closures can be provided. In case of MM and MCM, it can be specified whether the equations in terms of molecule numbers or concentrations are to be derived. A system of equations corresponding to the selected modeling approach is then derived, and provided as a MATLAB script file including the corresponding initial conditions. This symbolic representation is the basis for the rest of the simulation and analysis. CERENA extensively uses MATLAB Symbolic Math Toolbox for a variety of symbolic manipulations including symbolic differentiation, e.g., in the calculation of Jacobian matrices used to accelerate the numerical simulation.

The models can be exported to Data2Dynamics software [34] for parameter estimation and model selection. In addition, an optional intermediate MATLAB function can be generated for the numerical simulation of the symbolic equations using MATLAB ODE solver ode15s.

### Derivation of sensitivity equations and numerical integration

Forward and adjoint sensitivity equations for the selected model are derived based on the aforementioned symbolic representation. The complete symbolic representation can then be used to compile simulation files. CERENA uses CVODES and IDAS solvers of the SUNDIALS package [32] which are C implementations of solvers suited for efficient numerical integration of stiff ODEs and DAEs. Although the SUNDIALS package provides a MATLAB interface to the C solvers, the governing equations must be specified as MATLAB code, which adds an overhead to the overall computational cost of numerical simulation. To ensure efficiency, wrappers for CVODES and IDAS, which compile model-specific MEX-files from automatically generated native C code, have been implemented in CERENA. The compiled MEX-files are used for the numerical simulation of the system with given parameter values and time vector. Options for the numerical solvers and sensitivity analysis can be specified as inputs to the MEX simulation files. For efficient numerical simulation, essential capabilities of the SUNDIALS package can be exploited. The compiled MEX-files can be used for subsequent analysis.

### Stochastic simulations

The solvers based on differential equations are complemented by SSAs, e.g. to provide reference solutions. In the case of SSAs, realizations of the stochastic process are simulated. CERENA implements next-reaction methods for constant [2] and time-dependent propensities [28]. To the best of our knowledge, an implementation of the modified next-reaction method for systems with time-dependent propensities and delays is not available in other software packages. This method, implemented in CERENA, is exact for reaction networks with time-dependent propensities whose antiderivatives are available in closed-form. Otherwise, a numerical integration error is introduced. This error can be controlled by adjusting the integration error tolerance of the respective numerical solvers.

### Visualization

To facilitate the interpretation of the numerical simulation results, CERENA offers various visualization routines. Time courses for stochastic realizations, as well as mean and higher-order moments of species, can be plotted. Moreover, the full and marginal probabilities can be visualized for SSA, FSP and MCM. To illustrate the interaction between different network components and propagation of stochasticity, correlation and partial correlation maps, including movies of these maps over time, are provided.

## Application

In this Section, we present two biological models to demonstrate different features of CERENA, including the improved computational complexity. Furthermore, we exploit the comprehensiveness of CERENA to compare different approximative descriptions.

### Three-stage gene expression model

**Fig 3:**
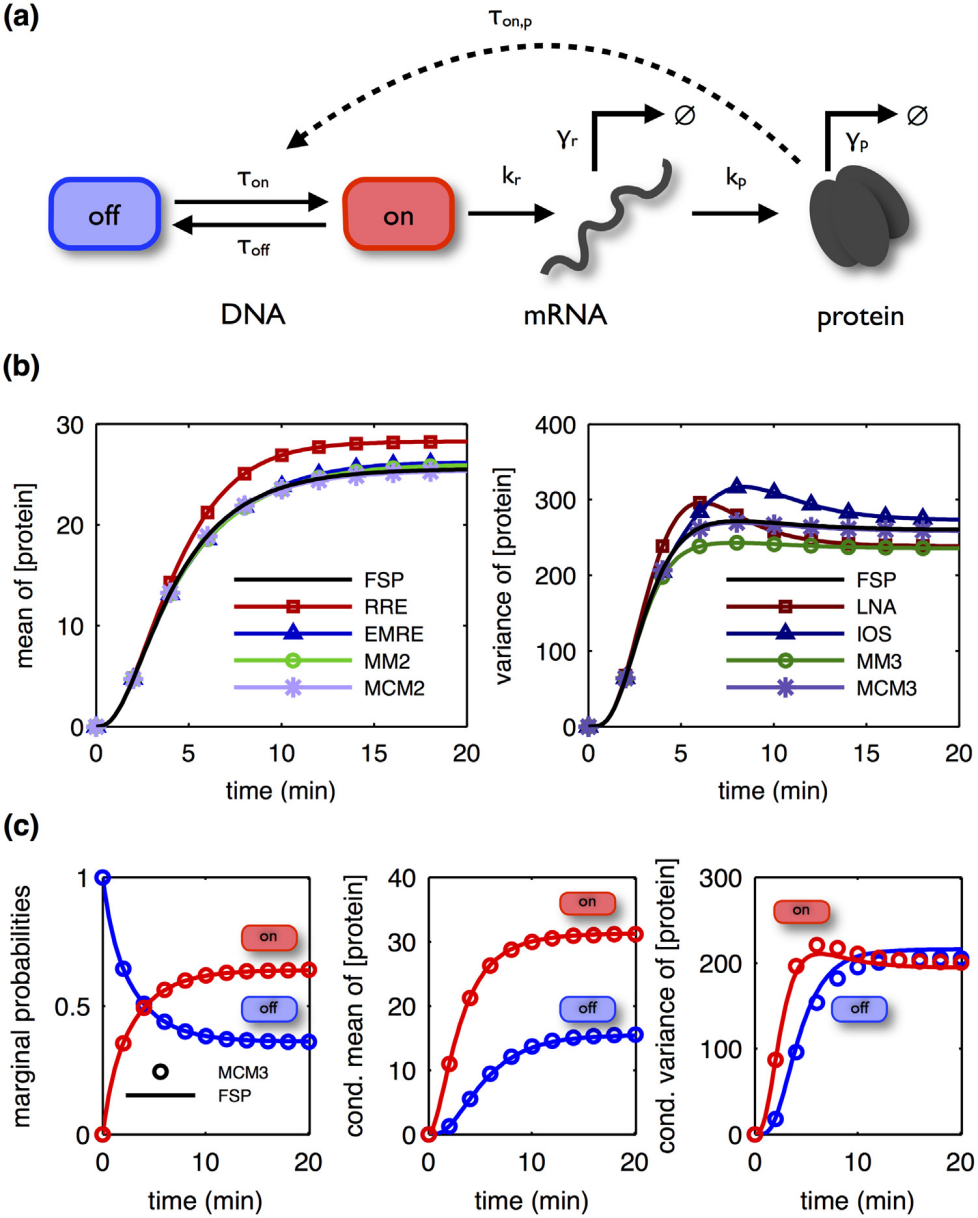
Simulation of the three-stage model of gene expression. (a) Schematic of the three-stage model of gene expression. (b) Mean (left) and variance (right) of the number of protein molecules obtained using different orders of SSE, MM and MCM. (c) Marginal probabilities of promotor states (left), the mean of protein molecule numbers conditioned on the promotor state (middle), and the variance of protein molecule numbers conditioned on the promotor state (right) predicted by MCM of order 3. (b,c) FSP results serve as the reference solution. Low dispersion closure was used for MM and MCM. MM2, MM3, MCM2 and MCM3 denote the second- and third-order MM and the second- and third-order MCM.

As the first example, we consider the generalized three-stage model of gene expression [3] depicted in Fig 3(a). This model includes a gene with a promotor switching between on- and off-states. Transcription of mRNA takes place if the promotor is in the on-state, and the transcribed mRNA can be translated into protein. The model also incorporates a protein-induced activation of the promoter which establishes a positive feedback loop. Protein and mRNA are subject to degradation. The combination of low-copy number species (the gene) and medium/high-copy number species (mRNA and protein) makes this model an interesting simulation test example.

#### Comprehensive comparison of approximation accuracy

The accuracy of various approximative descriptions is problem-specific, and therefore, comparisons of different descriptions for a process of interest is interesting in different applications. As demonstrated for this model, CERENA offers an easy-to-use framework for such a comprehensive comparison, thanks to its broad collection of simulation methods.

This process was implemented and simulated in CERENA for the parameter values given in S1 CERENA Documentation, Chapter 1, Table E. Fig 3(b) depicts the simulation results for the mean and the variance of the number of protein molecules obtained using various methods. All methods yield results which agree well with the reference solution, obtained using FSP. The RRE deviates the most from the reference solution. This behavior is expected, especially when the abundance of species is low, as RRE merely provides a macroscopic description of the stochastic process.

As mRNA is only transcribed if the promotor is in the on-state, the conditional distributions of mRNA and protein counts in the on- and off-states differ. These differences are captured by the MCM (Fig 3(c)), which provides information about the probability of different promotor states and the moments of the corresponding conditional distributions of the counts of mRNA and protein.

**Fig 4:**
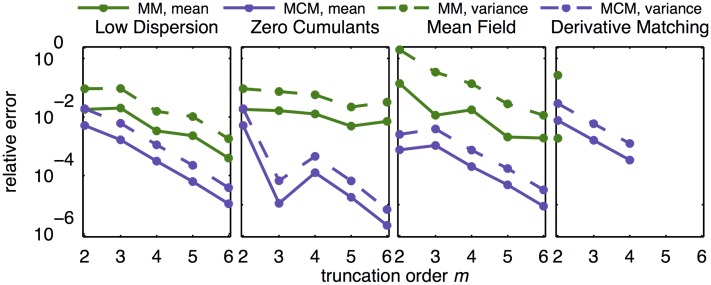
Approximation error of MM and MCM of various orders with various moment closures for the three-stage model of gene expression. Relative errors of mean and variance of the protein concentration at the steady state are depicted for different truncation orders and moment closures. The truncation order *m* means that moments up to order *m* are simulated. For moment orders and closures for which the numerical simulation could not be completed, i.e. derivative matching, no approximation error is reported.

The accuracy of different descriptions is quantified in terms of the relative errors of the mean and variance with respect to the FSP, e.g., |*μ*_MCM_−*μ*_FSP_|/*μ*_FSP_. Fig 4 displays the relative errors of MM and MCM close to steady state (t = 100), for various truncation orders and moment closures. For derivative matching closure, we find that the resulting ODE model cannot be simulated robustly as it diverges for several truncation orders. It is observed that the contribution of higher-order moments tends to enhance the simulation accuracy of lower-order moments. The influence of truncation order on the accuracy varies for different closure schemes.

#### Improved computational efficiency

**Fig 5:**
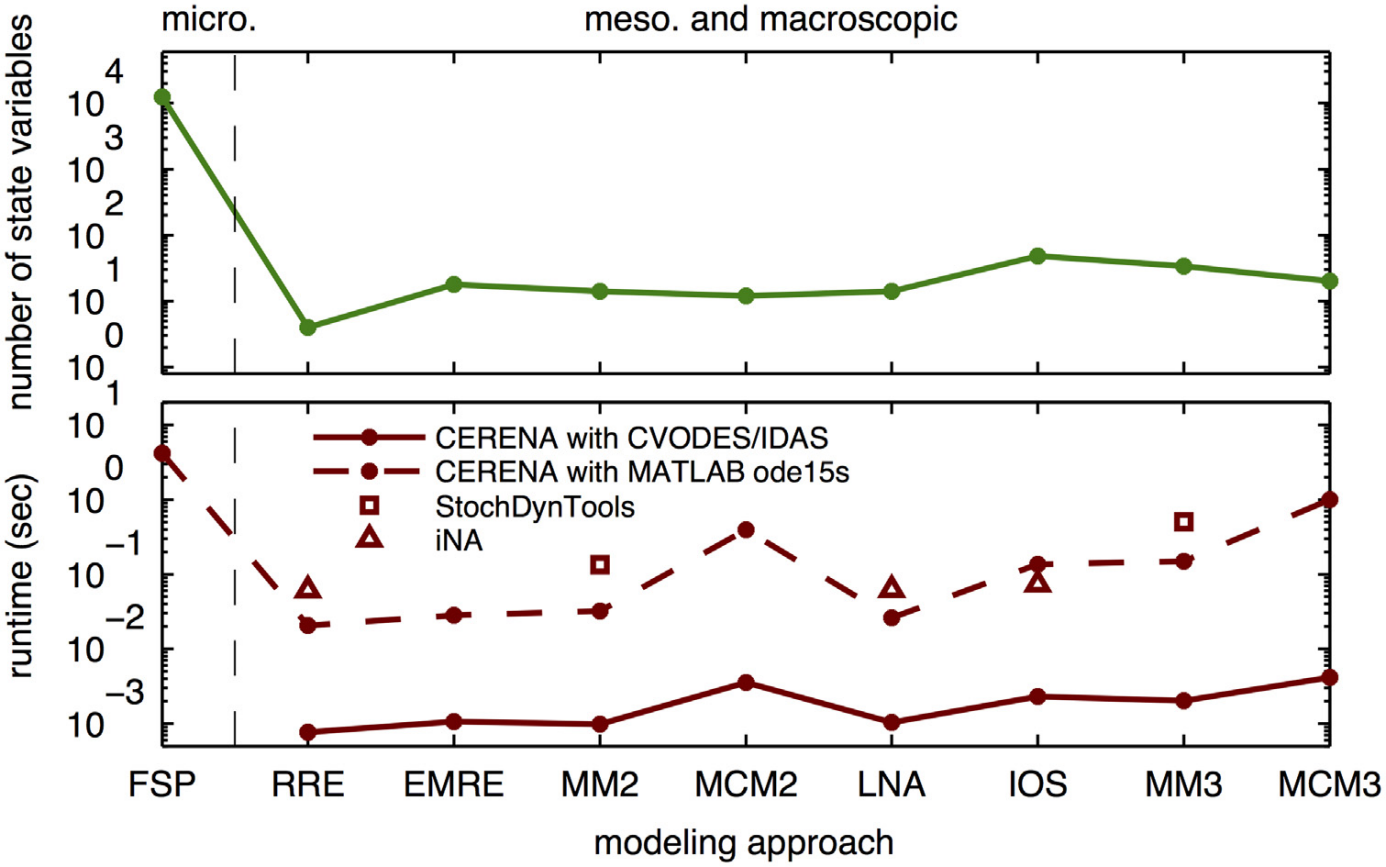
Complexity of different descriptions of the three-stage model of gene expression. Number of state-variables (top) and computation time (bottom). Runtimes are shown for the numerical simulation using CVODES/IDAS wrappers implemented in CERENA and MATLAB solver ode15s, as well as for StochDynTools. The computation times were calculated by averaging over at least 10 simulations. For MM and MCM low dispersion closure was used.

A key bottleneck in the analysis of stochastic chemical kinetics is the computational complexity of the numerical simulation. As the number of biochemical species or the approximation order increases, the system of differential equations to be solved becomes larger (Fig 5, top panel), indicating the need for efficient numerical simulation schemes. Since the FSP describes the full probability distribution, its system of equations is several orders of magnitude larger than the rest of the methods which merely capture a few moments of the probability distribution (Fig 5, top panel).

We assessed the computation time for implementations in CERENA and compared it to other packages/implementations (Fig 5, bottom panel). It is evident that the combination of CVODES and IDAS packages with corresponding wrappers in CERENA resulted in remarkable speedup, around 10–100 fold, compared to the use of standard MATLAB ODE-solvers, e.g., ode15s. Also, other toolboxes, e.g., StochDynTools and iNA, were outperformed by CERENA. A comparison across different methods reveals that the simulation of higher-order descriptions which possess more state-variables tends to be computationally more demanding than the simulation of lower-order descriptions.

### JAK-STAT signaling pathway

**Fig 6:**
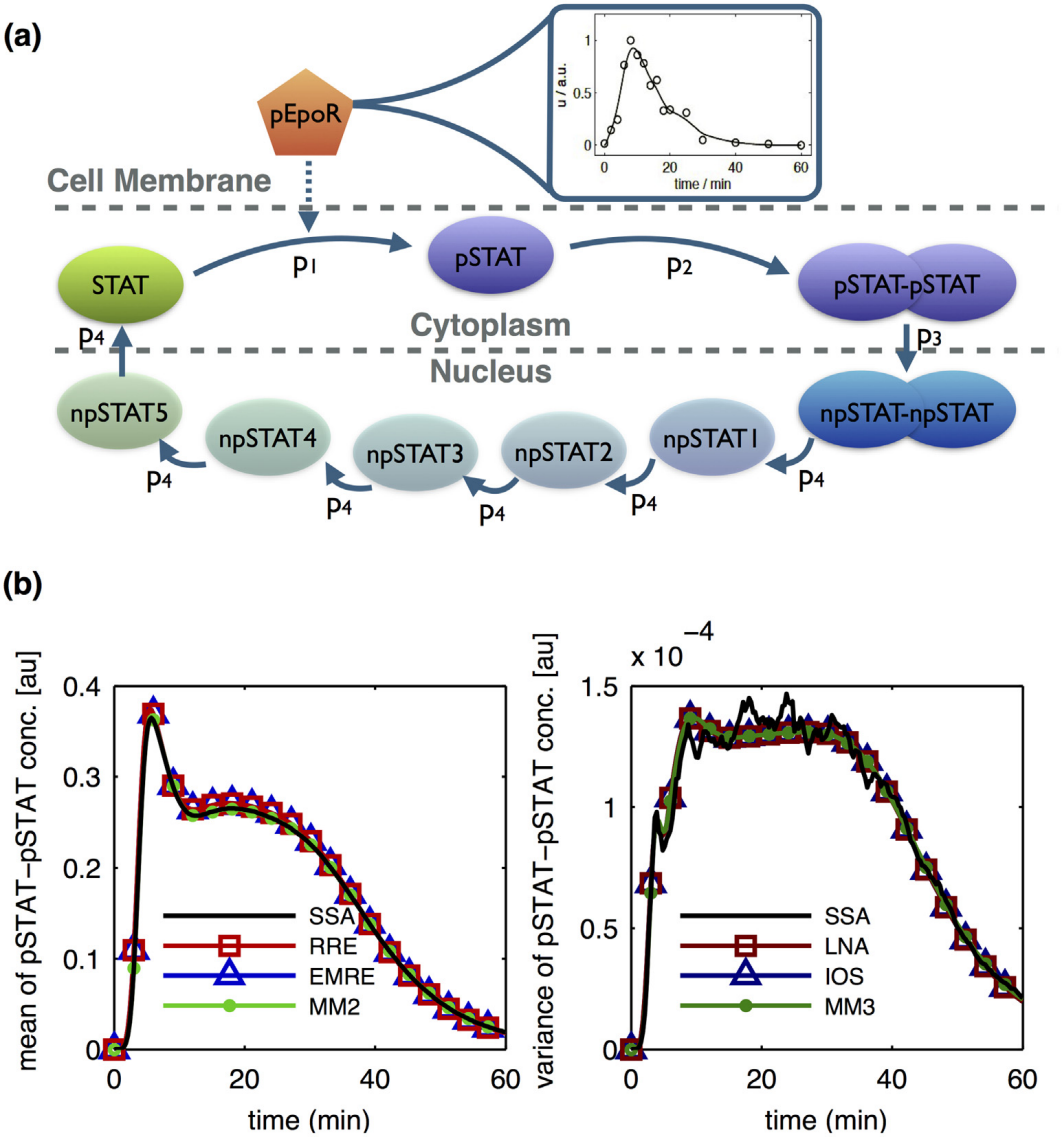
Simulation results for the JAK-STAT signaling pathway. (a) Schematic of the simplified JAK-STAT signaling pathway. The intermediate states npSTAT1 to npSTAT5 are used to model the delayed export of STAT from the nucleus. (b) The mean (left) and variance (right) of dimerized phosphorylated STAT concentration, obtained using several methods. SSA simulation results serve as the reference solution.

The second example studied using CERENA is a model of the JAK-STAT signaling pathway introduced by [35]. The model, sketched in Fig 6(a), describes the signaling cascade of STAT protein. Upon activation, the Epo receptor triggers the phosphorylation of cytoplasmic STAT. Dimerization and translocation of phosphorylated STAT into the nucleus, followed by a delayed export of STAT from the nucleus complete the loop. The time-dependent concentration of phosphorylated Epo receptor, [pEpoR], functions as an input to the system. The experimental data for the concentration of phosphorylated Epo receptor, cytoplasmic STAT and phosphorylated cytoplasmic STAT are available from previous studies [36].

The JAK-STAT signaling pathway is an interesting application example as it (i) includes two compartments, namely cytoplasm and nucleus, and (ii) involves a time-dependent propensity.

#### Simulation of multi-compartment systems with time-dependent propensities

We used CERENA to describe the dynamics of JAK-STAT signaling pathway for parameter values given in S1 CERENA Documentation, Chapter 8, Table A. As the copy numbers are relatively high in this pathway, MCM and FSP were not considered. To provide the reference solution, the modified next-reaction method for systems with time-dependent propensities implemented in CERENA was used which enabled handling of the time-dependent input. As seen in Fig 6(b), all methods showed the same qualitative behavior as the reference solution.

#### Comparison of sensitivity analysis methods

In previous studies, it was shown that the parameters of the JAK-STAT signaling pathway can be estimated efficiently for RRE [35] and EMRE and second-order MM descriptions [37]. These studies used gradient-based optimization methods with gradients being computed using forward sensitivity analysis. Here, we considered a weighted least-squares objective function as used by [37], and compared the performance of finite differences, forward and adjoint sensitivity analyses in gradient calculation for second- and third-order moment equations.

**Fig 7:**
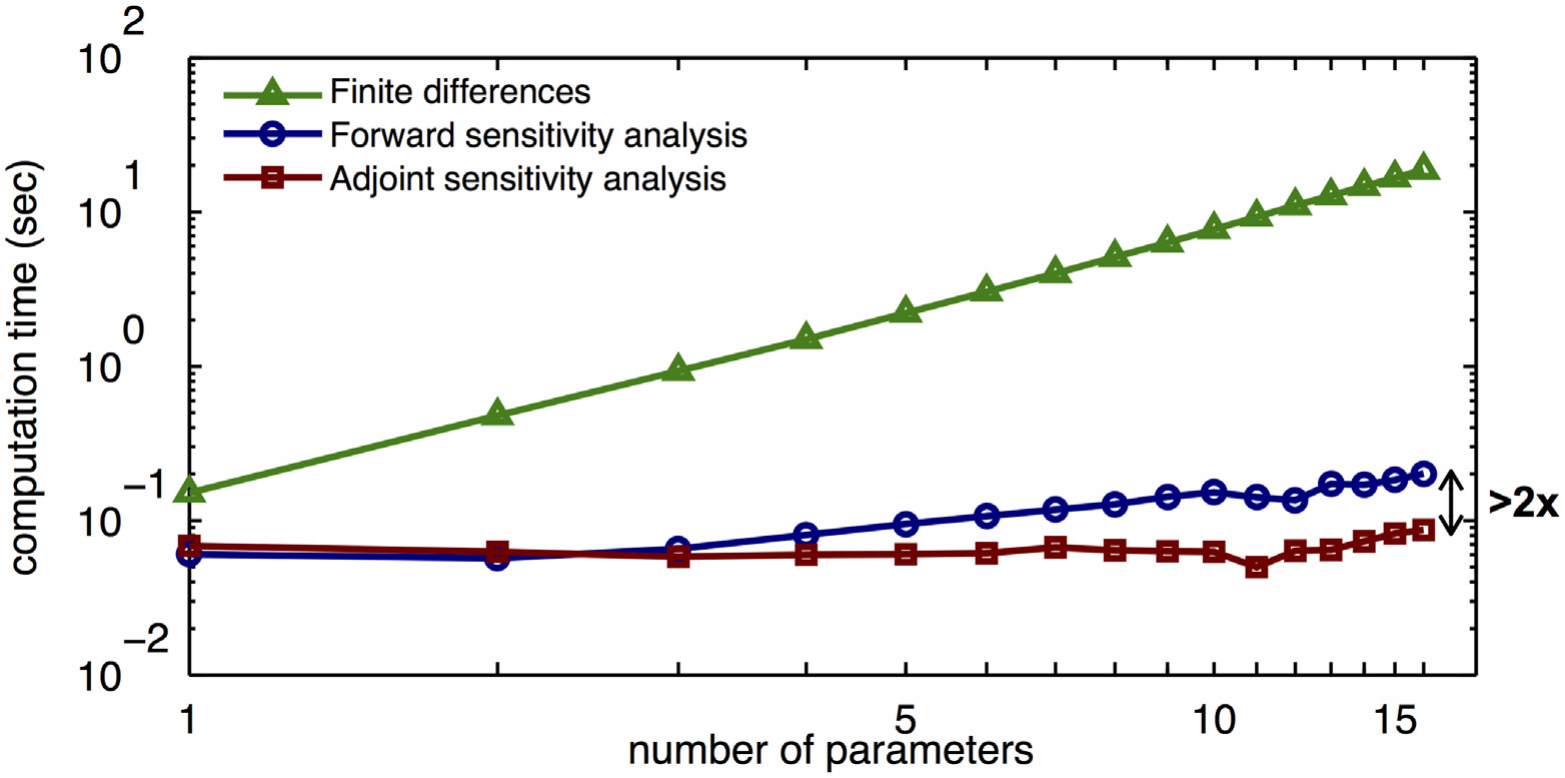
Computation time for different sensitivity analysis methods. The objective function gradient for MM2 simulation is evaluated for an increasing number of parameters. The computation times of finite differences, forward sensitivity analysis, and adjoint sensitivity analysis are shown.

We observed that, even for a small number of parameters, a gain in efficiency is achieved by using forward and adjoint sensitivity analysis methods instead of finite differences (Fig 7). Moreover, the adjoint sensitivity analysis has the best scalability with respect to the number of parameters.

## Discussion

A multitude of studies revealed the functional role of cell-to-cell variability in cellular mechanisms [5]. Hence, the analysis of cell-to-cell variability and its implications is crucial for a holistic understanding of biological systems, indicating the need for corresponding efficient simulation tools. In this work, we introduced CERENA, a user-friendly toolbox for the study of stochastic biological processes. CERENA offers a broad collection of simulation methods for micro-, meso- and macroscopic description of stochastic processes, rendering it unique compared to other software packages. In addition to various orders of the system size expansion and moment equations, the first implementation of the method of conditional moments is provided. CERENA attains generality not only method-wise, but also by imposing the least restrictions on the biological systems. Specifically, (regulatory) processes involving non-mass action kinetics, and/or time-dependent propensities can be analyzed. CERENA is one of the few packages to provide an SSA for the latter case. A key feature, distinguishing CERENA from all other packages for stochastic modeling, is the implementation of forward and adjoint sensitivity analyses for robust and efficient gradient calculations, especially in applications with high-dimensional parameter spaces. This enables feasible gradient-based optimization. To improve the computational efficiency, CERENA uses SUNDIALS solvers to compile numerical simulation MEX-files.

We used CERENA for detailed quantitative comparisons of different modeling approaches on models for three-stage gene expression and Epo-induced JAK-STAT signaling. These applications demonstrated that CERENA (i) offers suitable approximative methods for different biological regimes (or systems in different regimes of copy-numbers), and (ii) renders the comprehensive comparison of approximative descriptions and the subsequent selection straightforward. Also, the implementation of numerical solvers in CERENA proved to be significantly more efficient compared to other packages/implementations. For sensitivity analysis, a further acceleration was achieved by using forward and adjoint sensitivity analyses, with the latter possessing a superior scalability with respect to the number of parameters.

The current version of CERENA allows for the study of population-averaged and population snapshot data by providing time-dependent moments. To that end, a useful advancement could be realized by the integration of CERENA with sophisticated parameter estimation and model selection tools, such as ODE-constrained mixture modeling [38]. Complementarily, the moments obtained using MM, MCM and SSE could be used to compute a distribution approximation [39–41] to provide a more informative comparison with respect to SSA and FSP solutions. An automatic reconstruction of such approximative distributions could be incorporated in future releases of CERENA.

In conclusion, we have shown that CERENA is a comprehensive toolbox for stochastic modeling which maximizes both applicability and computational efficiency. This renders further studies of biological problems of realistic sizes feasible.

## Supporting Information

S1 CERENA Documentation The documentation of CERENA. This documentation includes a more detailed description of the modeling approaches implemented in CERENA, as well as elaborate instructions on using the CERENA toolbox.
(PDF)
