# Supplementary material for: CERENA: ChEmical REaction Network Analyzer—A Toolbox for the Simulation and Analysis of Stochastic Chemical Kinetics
Source: PLoS One. 2016 Jan 25;11(1):e0146732. doi: 10.1371/journal.pone.0146732 (PMC4726759; doi:10.1371/journal.pone.0146732)
Supplement: S1 CERENA Documentation — This documentation includes a more detailed description of the modeling approaches implemented in CERENA, as well as elaborate instructions on using the CERENA toolbox. (PDF) [file pone.0146732.s001.pdf]

User Documentation for CERENA: CERENA: ChEmical REaction Network Analyzer - A Toolbox for the Simulation and Analysis of Stochastic Chemical Kinetics

Atefeh Kazeroonian [1,2], Fabian Fröhlich [1,2], Andreas Raue [3],
Fabian J. Theis [1,2] and Jan Hasenauer [1,2*]

[1]Institute of Computational Biology, Helmholtz Zentrum München -
German Research Center for Environmental Health, Neuherberg, Germany,
[2]Department of Mathematics, Chair of Mathematical Modeling of Biological Systems,
Technische Universität München, Garching, Germany,
[3]Merrimack Pharmaceuticals Inc., Discovery Devision, Cambridge, MA 02139, USA

December 28, 2015

*to whom correspondence should be addressed.

Contents

1 Introduction to stochastic chemical kinetics **3**
1.1 Chemical reaction networks . . . 3
1.2 Example: The three-stage gene expression model . . . 5
1.3 Modeling and simulation approaches . . . 7
1.3.1 Stochastic Simulation Algorithm . . . 7
1.3.2 Finite State Projection . . . 8
1.3.3 Reaction Rate Equations . . . 8
1.3.4 System Size Expansion . . . 9
1.3.5 Method of Moments . . . 9
1.3.6 Method of Conditional Moments . . . 10
1.4 Sensitivity analysis . . . 12
1.4.1 Forward sensitivity equations . . . 12
1.4.2 Adjoint sensitivity equations . . . 13
1.5 Likelihood function evaluation . . . 13

2 CERENA startup **14**
2.1 Installation . . . 14
2.2 Requirements . . . 15

3 Setting up a model in CERENA **16**
3.1 Model definition file . . . 16
3.1.1 Example: The three-stage gene expression model . . . 20
3.2 SBML Import . . . 22
3.2.1 Example: The three-stage gene expression model . . . 22

4 Compiling Simulation Files **23**
4.1 Generation of simulation files . . . 23
`completeSystem()` . . . 24
`genmexp()` . . . 24
`cvodewrap()` . . . 24
`llhwrap()` . . . 24
`idawrap()` . . . 25
4.2 Specification of modeling approach . . . 25
4.2.1 System Size Expansion . . . 25
4.2.2 Method of Moments . . . 26
4.2.3 Method of Conditional Moments . . . 26
4.2.4 Finite State Projection . . . 28
4.2.5 Symbolic representation . . . 28
4.2.6 Other formats . . . 28
4.3 Compilation of simulation MEX-files . . . 28

4.3.1 Rewriting the initial conditions before the compilation of MEX-files 29
4.4 Example: The three-stage gene expression model 29
4.5 User-defined closure technique 30
4.5.1 Example: The three-stage gene expression model 31

5 Numerical simulation and sensitivity analysis **33**
5.1 Numerical simulation of the system 33
5.2 Numerical simulation with arbitrary initial conditions 35
5.3 Numerical simulation and likelihood evaluation 36
5.4 Sensitivity analysis 37
5.4.1 Forward sensitivity analysis 38
5.4.2 Adjoint sensitivity analysis 39
5.5 MATLAB-based simulation file 39
5.6 MATLAB-based FSP simulation 40

6 SSA simulation **42**
6.1 Description 42
6.1.1 Constant propensities 43
6.1.2 Time-dependent propensities 43
6.2 Example: The three-stage gene expression model 43

7 Post-processing and visualization of simulation results **45**
7.1 Visualization of trajectories 45
7.1.1 Visualization of MM simulation results 45
7.1.2 Visualization of SSE simulation results 46
7.1.3 Visualization of MCM simulation results 46
7.1.4 Visualization of FSP simulation results 47
7.1.5 Visualization of SSA simulation results 47
7.1.6 Visualization of several simulation results 48
7.1.7 General options 48
7.2 Visualization of correlation and partial correlation 48
7.2.1 Correlation map 48
7.2.2 Partial correlation map 49
7.3 Example: The three-stage gene expression model 49

8 Further examples **55**
8.1 JAK-STAT signaling pathway 55
8.2 PapI regulation model 58
8.3 A gene cascade network 58

Chapter 1

Introduction to stochastic chemical kinetics

In this chapter, we briefly explain the fundamental concepts of the stochastic reaction kinetics and the modeling approaches used for the simulation of such kinetics.

1.1 Chemical reaction networks

Consider a biological system comprising a chemical reaction network. The kinetics of such a network is usually modeled by a system of *chemical species* which undergo a set of *chemical reactions*. A *chemical species* represents an ensemble of molecules that are chemically identical, such as RNA molecules. A *chemical reaction* is a process in which chemical species are produced, degraded or converted into other chemical species. Accordingly, a chemical reaction network can be described as a system of:

- n_s chemical species, $S_1, S_2, \cdots, S_{n_s}$, and
- n_r chemical reactions, $R_1, R_2, \cdots, R_{n_r}$,

where the jth chemical reaction, R_j, is a process as below:

$$R_j : \sum_{i=1}^{n_s} \nu_{i,j}^{-} S_i \xrightarrow{k_j} \sum_{i=1}^{n_s} \nu_{i,j}^{+} S_i$$

where k_j denotes the kinetic constant of reaction R_j. The coefficient $\nu_{i,j}^{-} \in \mathbb{N}_0$ denotes the stoichiometric coefficient of species S_i as a reactant in reaction R_j, and is defined as the number of S_i molecules consumed in reaction R_j. Similarly, the coefficient $\nu_{i,j}^{+} \in \mathbb{N}_0$ denotes the stoichiometric coefficient of species S_i as a product in reaction R_j, and is defined as the number of S_i molecules produced in reaction R_j. The overall stoichiometric coefficient of species S_i in reaction R_j, $\nu_{i,j} = \nu_{i,j}^{+} - \nu_{i,j}^{-} \in \mathbb{Z}$ is defined as the net change in the count of S_i when reaction R_j takes place. The overall stoichiometry of the reaction R_j is then given by the vector $\nu_j = (\nu_{1,j}, \nu_{2,j}, \cdots, \nu_{n_s,j})^T \in \mathbb{Z}^{n_s}$.

The state of the chemical reaction network at time t is represented by a vector $\mathbf{X_t} = (X_{1,t}, X_{2,t}, \ldots, X_{n_s,t}) \in \mathbb{N}_0^{n_s}$, where $X_{i,t}$ denotes the count of species S_i at time t, and remains constant as long as no reactions occur. Upon firing of the reaction R_j, the state transition $\mathbf{X_t} \to \mathbf{X_t} + \nu_j$ occurs. The statistics of the time until the firing of next reaction, as well as the index of the next reaction, are determined by the *reaction propensities*. The *propensity* of reaction R_j, $a_j(\mathbf{X}_t) : \mathbb{N}_0^{n_s} \to \mathbb{R}_+$ is a function of the state of the system. If we assume

Table A: Reaction propensities according to the law of mass action.

Reaction Order	Reaction Type	Propensity
0	$\emptyset \xrightarrow{k_j}$ product	k_j
1	$S_i \xrightarrow{k_j}$ product	$k_j X_i$
2	$S_i + S_l \xrightarrow{k_j}$ product	$k_j X_i X_l$
2	$S_i + S_i \xrightarrow{k_j}$ product	$\frac{1}{2} k_j X_i (X_i - 1)$

that the kinetics follow the law of mass action, the reaction propensities are determined by the order and the type of reaction as given in Table A. Here, k_j denotes the kinetic constant of reaction R_j.

The reaction kinetics need not necessarily follow the law of mass action, and can be of more complicated forms. For example, the Mechaelis-Menten kinetics describes the rate of the enzymatic reactions as $\frac{V_{\max}[X]}{K_M+[X]}$, where $V_{\max}$ and K_M are constants, and $[X]$ denotes the concentration of a substrate.

Microscopic and macroscopic propensities and parameters.

The propensities $a_j(\mathbf{X_t})$, as given in Table A, describe the changes in the number of molecules of species, i.e., the changes in the state vector $\mathbf{X_t}$. Therefore, these propensities, as well as the kinetic constants k_j, are independent from the volume of the compartment containing the chemical reaction network. We refer to them as *microscopic propensities* and *microscopic parameters.*

In some cases, for instance in reaction rate equations (see Section 1.3.3), one may be interested in the concentration of species instead of their counts. In this case, the counts of species can be converted to the corresponding concentrations by the conversion below

$$X_i' = \frac{X_i}{\Omega}, \tag{1.1}$$

where X_i' denotes the concentration of species S_i and Ω denotes the volume of the compartment.

Attention! Formula (1.1) yields the *number concentration* i.e. the number of molecules per volume. To calculate the *molar concentration*, one should use $X_{m,i}' = \frac{X_i}{N_A\,\Omega}$, where $N_A \approx 6.022 \times 10^{23}$ mol^{-1} is Avogadro constant. If the user is interested in *molar concentration*, they should provide $N_A\,\Omega$ as the volume of the compartment instead of Ω.

To obtain the propensities for the concentrations, we first need to substitute the counts X_i in microscopic propensities by concentrations X_i'. In addition, to account for the fact that the propensity describes the changes in the concentrations, we need to divide the microscopic propensity by the volume Ω*. For example, consider the propensity of the second-order reaction in Table A, $k_j X_i X_l$. The corresponding propensity in terms of concentrations is obtained as

$$a_j'(\mathbf{X}') = \frac{1}{\Omega} k_j (\Omega X_i')(\Omega X_l') = k_j \Omega X_i' X_l'.$$

$a_j'(\mathbf{X}_t')$ is mostly known as the *macroscopic rate function*. If we introduce a *macroscopic* parameter $k_j' = k_j \Omega$, we retrieve the previous form given in Table A for the macroscopic rate function:

$$a_j'(\mathbf{X}') = k_j' X_i' X_l'.$$

*For an auto-dimerization reaction, an additional change is required to derive the macroscopic propensity. See Formula (1.2) and Table C for more details.

Table B: Relationship between the microscopic and macroscopic parameters according to the law of mass action.

Reaction Order	Reaction Type	Microscopic Kinetic Constant	Macroscopic Kinetic Constant
0	$\emptyset \xrightarrow{k_j}$ product	k_j	$k'_j = \frac{k_j}{\Omega}$
1	$S_i \xrightarrow{k_j}$ product	k_j	$k'_j = k_j$
2	$S_i + S_l \xrightarrow{k_j}$ product	k_j	$k'_j = k_j\Omega$
2	$S_i + S_i \xrightarrow{k_j}$ product	k_j	$k'_j = k_j\Omega$

Table C: Macroscopic propensities according to the law of mass action.

Reaction Order	Reaction Type	Propensity
0	$\emptyset \xrightarrow{k'_j}$ product	k'_j
1	$S_i \xrightarrow{k'_j}$ product	$k'_j X'_i$
2	$S_i + S_l \xrightarrow{k'_j}$ product	$k'_j X'_i X'_l$
2	$S_i + S_i \xrightarrow{k'_j}$ product	$k'_j (X'_i)^2$

In many models, including the reaction rate equations (see Section 1.3.3), the macroscopic parameters are used instead of the microscopic parameters. Unlike their microscopic counterparts, the macroscopic parameters and macroscopic rate functions depend on the volume Ω. The conversion of the microscopic parameters to macroscopic parameters depends on the order of the reaction, and can be done as explained above. Table B provides the relationship between the macroscopic and microscopic parameters for several reaction types.

We noted earlier that the macroscopic rate functions in terms of macroscopic parameters, $a'_j(\mathbf{X}'_t)$, follow the forms given in Table A. Only in the case of an auto-dimerization reaction, $S_i + S_i \xrightarrow{k_j}$ product, the macroscopic rate function is given by

$$a'_j(\mathbf{X}') = k_j(X'_i)^2. \tag{1.2}$$

Attention! To avoid confusions and potential mistakes, CERENA only allows the user to provide the microscopic propensities together with microscopic parameters (as in Table A), or the macroscopic rate functions together with macroscopic parameters (as in Table C). See Section 3.1 for further instructions. If needed, the user can refer to Table B to obtain the correct parameter values for the desired propensities.

1.2 Example: The three-stage gene expression model

In the following, we illustrate the aforementioned notions using an example. We choose the three-stage gene expression model [1] and extend it by a feedback loop to introduce a nonlinearity. A schematic of the model is depicted in Figure 1.1. This system will be used in later sections to demonstrate the capabilities of CERENA.

This model includes a gene with a promotor switching between on- and off-states. Transcription of mRNA takes place if the promotor is in the on-state, and the transcribed mRNA can be translated into protein. The model also incorporates a protein-induced activation of the promoter which establishes a positive feedback loop. The protein and mRNA are subject to degradation.

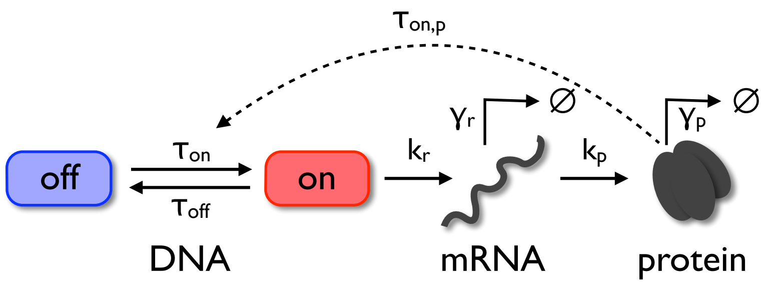

Figure 1.1: Schematic of the three-stage gene expression model.

Table D: Chemical reactions of the three-stage gene expression example.

Reactions	Microscopic Propensity	Macroscopic Propensity	Parameters
$R_1:\ \mathrm{DNA_{off}} \overset{\tau_{\rm on}}{\to} \mathrm{DNA_{on}}$	$\tau_{\rm on}\, X_{\rm DNA_{off}}$	$\tau'_{\rm on}\, X'_{\rm DNA_{off}}$	$\tau'_{\rm on} = \tau_{\rm on}$
$R_2:\ \mathrm{DNA_{on}} \overset{\tau_{\rm off}}{\to} \mathrm{DNA_{off}}$	$\tau_{\rm off}\, X_{\rm DNA_{on}}$	$\tau'_{\rm off}\, X'_{\rm DNA_{on}}$	$\tau'_{\rm off} = \tau_{\rm off}$
$R_3:\ \mathrm{DNA_{on}} \overset{k_r}{\to} \mathrm{DNA_{on}} + \mathrm{mRNA}$	$k_r\, X_{\rm DNA_{on}}$	$k'_r\, X'_{\rm DNA_{on}}$	$k'_r = k_r$
$R_4:\ \mathrm{mRNA} \overset{\gamma_r}{\to} \emptyset$	$\gamma_r\, X_{\rm mRNA}$	$\gamma'_r\, X'_{\rm mRNA}$	$\gamma'_r = \gamma_r$
$R_5:\ \mathrm{mRNA} \overset{k_p}{\to} \mathrm{mRNA} + \mathrm{protein}$	$k_p\, X_{\rm mRNA}$	$k'_p\, X'_{\rm mRNA}$	$k'_p = k_p$
$R_6:\ \mathrm{protein} \overset{\gamma_p}{\to} \emptyset$	$\gamma_p\, X_{\rm protein}$	$\gamma'_p\, X'_{\rm protein}$	$\gamma'_p = \gamma_p$
$R_7:\ \mathrm{protein} + \mathrm{DNA_{off}} \overset{\tau^{\rm p}_{\rm on}}{\to} \mathrm{protein} + \mathrm{DNA_{on}}$	$\tau^{\rm p}_{\rm on}\, X_{\rm DNA_{off}}\, X_{\rm protein}$	$\tau'^{\rm p}_{\rm on}\, X'_{\rm DNA_{off}}\, X'_{\rm protein}$	$\tau'^{\rm p}_{\rm on} = \Omega\, \tau^{\rm p}_{\rm on}$

The chemical species of this model are:

S_1 : $\mathrm{DNA_{on}}$ (promoter on-state), S_2 : $\mathrm{DNA_{off}}$ (promoter off-state), S_3 : mRNA, S_4 : protein.

Table E: Parameter values and initial conditions of the three-stage gene expression example.

$\tau_{\rm on}$	$\tau_{\rm off}$	k_r	γ_r	k_p	γ_p	$\tau^{\rm p}_{\rm on}$	Ω	$X_{\rm DNA_{off}}(0)$	$X_{\rm DNA_{on}}(0)$	$X_{\rm mRNA}(0)$	$X_{\rm protein}(0)$
0.3	0.3	10	1	4	1	0.015	1	1	0	0	0

The state vector of the system, $\mathbf{X} = (X_{\rm DNA_{off}}, X_{\rm DNA_{on}}, X_{\rm mRNA}, X_{\rm protein})$, represents the counts of species. Table D presents the list of the chemical reactions of this model, together with their propensities. According to the reactions in Table D, the stoichiometry matrix of the system is given as:

$$
\begin{array}{c} \\ X_{\rm DNA_{off}} \\ X_{\rm DNA_{on}} \\ X_{\rm mRNA} \\ X_{\rm protein} \end{array}
\begin{array}{c}
\begin{array}{ccccccc} R_1 & R_2 & R_3 & R_4 & R_5 & R_6 & R_7 \end{array} \\
\begin{pmatrix} -1 & 1 & 0 & 0 & 0 & 0 & -1 \\ 1 & -1 & 0 & 0 & 0 & 0 & 1 \\ 0 & 0 & 1 & -1 & 0 & 0 & 0 \\ 0 & 0 & 0 & 0 & 1 & -1 & 0 \end{pmatrix}
\end{array},
$$

where the $i^{\rm th}$ row represents the count of species S_i, and the $j^{\rm th}$ column represents reaction R_j.

In this documentation, we use the parameter values and initial conditions given in Table E to simulate the three-stage gene expression example using different modeling approaches.

1.3 Modeling and simulation approaches

A chemical reaction network, comprising of n_s chemical species, $S_1, \dots, S_{n_s}$, and n_r chemical reactions, $R_1, \dots, R_{n_r}$, is described using a continuous-time Markov chain (CTMC) [2]. The state of this CTMC is represented by the state vector $\mathbf{X} = (X_1, \dots, X_{n_s})^T$, where X_i denotes the count of species S_i. As explained in Section 1.1, the state $\mathbf{X}$ changes by the firing of chemical reactions.

The probability of observing the CTMC at a particular state $\mathbf{x} = (x_1, \dots, x_{n_s})^T$ at time t is denoted by $p(\mathbf{x}|t)$. The time evolution of the probability distribution $p(\mathbf{x}|t)$ is governed by the CME, which is a system of ordinary differential equations (ODEs) for every possible state $\mathbf{x}$:

$$\frac{\partial}{\partial t} p(\mathbf{x}|t) = \sum_{j=1}^{n_r} \Big(a_j(\mathbf{x} - \nu_j) p(\mathbf{x} - \nu_j|t) - a_j(\mathbf{x}) p(\mathbf{x}|t) \Big). \tag{1.3}$$

As solving the CME is mostly infeasible due to the large or infinite number of states $\mathbf{x}$, various approximative methods have been developed. Several methods concentrate on the full distribution $p(\mathbf{x}|t)$ to provide a microscopic description. For mesoscopic and macroscopic descriptions, there exist several methods that focus on representing the solution of the CME in terms of its statistical moments, i.e.,

$$\begin{aligned} \text{mean} \quad & \mathbf{m} = (m_1, \dots, m_{n_s})^T = \sum_{\mathbf{x}} \mathbf{x} p(\mathbf{x}|t), \\ \text{covariance} \quad & \mathbf{C} = \sum_{\mathbf{x}} (\mathbf{x} - \mathbf{m})(\mathbf{x} - \mathbf{m})^T p(\mathbf{x}|t), \\ \text{higher-order moments} \quad & \mathbf{C_I} = \sum_{\mathbf{x}} (\mathbf{x} - \mathbf{m})^{\mathbf{I}} p(\mathbf{x}|t). \end{aligned} \tag{1.4}$$

Here, we use the following product notation:

$$(\mathbf{x} - \mathbf{m})^{\mathbf{I}} := \prod_{i=1}^{n_s} (x_i - m_i)^{I_i}, \tag{1.5}$$

where $\mathbf{I} = (I_1, \dots, I_{n_s})$ is a vector of non-negative integers. The order of the moment $\mathbf{C_I}$ is given by $M = \sum_{i=1}^{n_s} I_i$.

The microscopic, mesoscopic, macroscopic and hybrid methods implemented in CERENA, are briefly introduced in the following.

1.3.1 Stochastic Simulation Algorithm

SSAs generate statistically representative sample paths of the CTMC [3]. A next-reaction SSA method is implemented in CERENA. In this method, starting from an initial condition $\mathbf{X_0} = (X_{1,0}, \dots, X_{n_s,0})^T$, randomly sampled from an initial distribution, the time until the next reaction and the index of the next reaction to fire are sampled from their corresponding distributions. The state vector $\mathbf{X}$ is then updated by $\mathbf{X}_\tau \to \mathbf{X}_{\tau-} + \nu_j$, where j is the index of the next reaction to fire, and τ is the time of the firing. In this way, sample paths of the stochastic process are generated for the time interval of interest. An estimate to the probability distribution $p(\mathbf{x}|t)$ is given by the frequency of sample paths that occupy state $\mathbf{x}$ at time t. To estimate the moments of the process, Monte-Carlo integration can be performed. For example, the mean

and variance of the process are approximated by

$$\mathbf{m}(t) = \frac{1}{N}\sum_{l=1}^{N}\mathbf{x}_l(t),$$
$$\mathbf{C}(t) = \frac{1}{N-1}\sum_{l=1}^{N}\Big(\mathbf{x}_l(t)-\mathbf{m}(t)\Big)\Big(\mathbf{x}_l(t)-\mathbf{m}(t)\Big)^T, \tag{1.6}$$

where N is the number of generated sample paths, and $\mathbf{x}_l(t)$ is the state of the CTMC at time t in the lth sample path. While the estimators for probability distribution and moments are unbiased and converge, the sample-sizes required to obtain low-variance estimates are generally large, rendering SSA-based methods computationally demanding.

In the case of time-dependent propensities, CERENA offers a modified next-reaction method [4], which takes into account the exact evolution of the time-dependent propensities in between the reaction firings.

1.3.2 Finite State Projection

To enable a direct approximation of $p(\mathbf{x}|t)$, the FSP [5] considers a finite subspace of the CME, $\Psi \subset \mathbb{N}_0^{n_s}$, which only contains states with non-negligible probabilities $p(\mathbf{x}|t)$. All other states $\mathbf{x} \notin \Psi$ are lumped into a single sink state which only absorbs probability from states $\mathbf{x} \in \Psi$, but does not return any probability back to the system. In this way, the set of ODEs for the probabilities of states $\mathbf{x} \in \Psi$, derived from the CME, yield a lower bound for $p(\mathbf{x}|t)$. This lower bound, denoted by $\underline{p}(\mathbf{x}|t)$, is the solution of the following ODE system:

$$\forall \mathbf{x} \in \Psi : \frac{\partial}{\partial t}\underline{p}(\mathbf{x}|t) = \sum_{\substack{j=1 \\ \mathbf{x}-\nu_j \in \Psi}}^{n_r} a_j(\mathbf{x}-\nu_j)\underline{p}(\mathbf{x}-\nu_j|t) - \sum_{j=1}^{n_r} a_j(\mathbf{x})\underline{p}(\mathbf{x}|t). \tag{1.7}$$

Growing the state-space of FSP, Ψ, decreases the approximation error at the cost of increased computational complexity. In FSP simulations, the sum of the probabilities remained in the system, $\sum_{\mathbf{x}\in\Psi}\underline{p}(\mathbf{x}|t)$, is an indicator of the approximation error by truncating the state space. For details we refer to [5].

1.3.3 Reaction Rate Equations

The RRE is the most commonly used modeling approach for biochemical reaction networks. It constitutes a system of ODEs for the time evolution of the mean of the stochastic process in the macroscopic limit. For reaction networks with constant and linear propensities, i.e. those with only zero- or monomolecular reactions, the solution of the RRE is exactly the mean of the stochastic process. For reaction networks with nonlinear propensities, the RRE prediction can be considerably different from the true mean of the process since it neglects the stochastic effects. In such cases, the solution of RRE is reflective of the true mean of the stochastic process only in the limit of large molecule numbers [7].

The RRE equations for the time evolution of the mean of species can be derived by multiplying the stoichiometric matrix by the vector of reaction propensities (see Section 1.1):

$$\frac{\partial \mathbf{m}(t)}{\partial t} = \begin{pmatrix} \frac{\partial m_1(t)}{\partial t} \\ \frac{\partial m_2(t)}{\partial t} \\ \vdots \\ \frac{\partial m_{n_s}(t)}{\partial t} \end{pmatrix} = \begin{pmatrix} \nu_{1,1} & \nu_{1,2} & \cdots & \nu_{1,n_r} \\ \nu_{2,1} & \nu_{2,2} & \cdots & \nu_{2,n_r} \\ \vdots & \vdots & \ddots & \vdots \\ \nu_{n_s,1} & \nu_{n_s,2} & \cdots & \nu_{n_s,n_r} \end{pmatrix} \begin{pmatrix} a_1(\mathbf{m}(t)) \\ a_2(\mathbf{m}(t)) \\ \vdots \\ a_{n_r}(\mathbf{m}(t)) \end{pmatrix}.$$

Here, $m_i(t)$ denotes the mean of species S_i at time t, $\nu_{i,j}$ denotes the stoichiometric coefficient of species S_i in reaction R_j, and $a_j(\mathbf{m}(t))$ denotes the propensity of reaction R_j evaluated at the mean $\mathbf{m}(t)$.

1.3.4 System Size Expansion

For a systematic approximation of the dynamics of mesoscopic systems, the SSE has been introduced [6]. The SSE is a power series expansion of the CME in the inverse volume of the system. Depending on the order of the SSE, different ODE systems approximating the moments of the CME solution are obtained. The lowest-order approximation for the mean reproduces the aforementioned RRE. For the covariance, the lowest-order approximation yields the well-known linear noise approximation (LNA) [6], whose validity has been studied in [7] for different classes of reaction systems. For CMEs with unimodal solutions, the LNA can be interpreted as a Gaussian approximation of the probability density function of the CME. Higher-order corrections for the mean and covariance yield the effective mesoscopic rate equation (EMRE) [8] and the inverse omega square (IOS) approximation [9]. These methods tend to be more accurate for systems of small and medium volumes [10]. For the SSE equations, we refer to [8] and [9].

1.3.5 Method of Moments

The method of moments (MM) [11] is conceptually similar to the SSE in that it also sets a framework for describing the moments of the solution of CME. Following the definition of moments, a system of ODEs for the exact time evolution of the moments, which constitutes the moment equations, can be derived from the CME.

Given that the reactions are at most bimolecular, the MM equations for the mean, (co)variance and higher-order moments can be written as

$$
\begin{aligned}
\frac{\partial m_i(t)}{\partial t} &= \sum_{j=1}^{n_r} \nu_{i,j} \left(a_j(\mathbf{m}(t)) + \frac{1}{2} \sum_{k_1,k_2} \frac{\partial^2 a_j(\mathbf{m}(t))}{\partial x_{k_1} \partial x_{k_2}} C_{k_1 k_2}(t) \right), \\
\frac{\partial C_{[I_1,\cdots,I_{n_s}]}(t)}{\partial t} &= \sum_{j=1}^{n_r} a_j(\mathbf{m}(t)) \sum_{\substack{l_1,l_2,\cdots,l_{n_s} \\ l_1+l_2+\cdots+l_{n_s} \neq M}} \binom{I_1}{l_1} \cdots \binom{I_{n_s}}{l_{n_s}} \nu_{1,j}^{I_1-l_1} \cdots \nu_{n_s,j}^{I_{n_s}-l_{n_s}} C_{[l_1,\cdots,l_{n_s}]}(t) \\
&\quad + \sum_{j=1}^{n_r} \sum_{k} \frac{\partial a_j(\mathbf{m}(t))}{\partial x_k} \sum_{\substack{l_1,l_2,\cdots,l_{n_s} \\ l_1+l_2+\cdots+l_{n_s} \neq M}} \binom{I_1}{l_1} \cdots \binom{I_{n_s}}{l_{n_s}} \nu_{1,j}^{I_1-l_1} \cdots \nu_{n_s,j}^{I_{n_s}-l_{n_s}} C_{[l_1,\cdots,l_k+1,\cdots,l_{n_s}]}(t) \\
&\quad + \frac{1}{2} \sum_{j=1}^{n_r} \sum_{k_1,k_2} \frac{\partial^2 a_j(\mathbf{m}(t))}{\partial x_{k_1} \partial x_{k_2}} \sum_{\substack{l_1,l_2,\cdots,l_{n_s} \\ l_1+l_2+\cdots+l_{n_s} \neq M}} \binom{I_1}{l_1} \cdots \binom{I_{n_s}}{l_{n_s}} \nu_{1,j}^{I_1-l_1} \cdots \nu_{n_s,j}^{I_{n_s}-l_{n_s}} C_{[l_1,\cdots,l_{k_1}+1,\cdots,l_{k_2}+1,\cdots,l_{n_s}]}(t) \\
&\quad - \sum_{k=1}^{n_s} I_k \frac{\partial m_k(t)}{\partial t} C_{[I_1,\cdots,I_k-1,\cdots,I_{n_s}]}(t).
\end{aligned}
\tag{1.8}
$$

Here, $C_{ij}(t)$ denotes the covariance of species S_i and S_j, $C_{i_1 k_1 k_2}(t)$ denotes the third-order moment of species S_{i_1}, S_{k_1} and S_{k_2}, and $C_{[I_1,\cdots,I_{n_s}]}(t)$ denotes the Mth-order moment with $M = I_1 + I_2 + \cdots + I_{n_s}$. For details of derivation, we refer to [11] and [12]. The equations for moments of order M generally depend on moments of order $M+1$ and higher. For example in (1.8), the time evolution of the mean depends on the covariances. Therefore, moment closures are applied yielding a closed set of approximative moment equations.

Moment closure schemes approximate the higher-order moments as functions of lower-order moments by making specific assumptions about the distribution. Commonly used closure techniques include [13]

- Low dispersion closure: Assuming higher-order central moments are zero.
- Mean field closure: Assuming independence of variables (i.e. species concentrations).

- Zero cumulants closure: Assuming the distribution is similar to a normal distribution.
- Derivative matching closure: Assuming the distribution is similar to a log-normal distribution.

The accuracy of these closures is problem-specific.

1.3.6 Method of Conditional Moments

A hybrid approach for the approximation of the CME solution is provided by the method of conditional moments (MCM) [14]. The MCM combines a microscopic description of low copy-number species with a moment-based description of high copy-number species. Since stochastic fluctuations are more dominant for low copy-number species, marginal probability densities for these species are determined. The distributions of high-copy number species are merely described in terms of their moments, conditioned on the state of the low-copy number species. If we decompose the state vector $\mathbf{X}_t$ into two vectors $\mathbf{Y}_t$ and $\mathbf{Z}_t$ such that $\mathbf{Y}_t$ represents the state of low copy-number species and $\mathbf{Z}_t$ represents the state of high copy-number species, then the state probability $p(\mathbf{x}|t)$ is written as the joint probability

$$p(\mathbf{x}|t) = p(\mathbf{y}, \mathbf{z}|t) = p(\mathbf{z}|\mathbf{y}, t)p(\mathbf{y}|t). \tag{1.9}$$

$p(\mathbf{y}|t)$ denotes the probability of $\mathbf{Y}_t = \mathbf{y}$, and $p(\mathbf{z}|\mathbf{y}, t)$ denotes the conditional probability of $\mathbf{Z}_t = \mathbf{z}$ given that $\mathbf{Y}_t = \mathbf{y}$.

Accordingly, the state variables of the MCM are defined as

- Marginal probabilities of low copy-number species:

$$p(\mathbf{y}|t) = \sum_{\mathbf{z}\geq\mathbf{0}} p(\mathbf{y}, \mathbf{z}|t)$$

- Conditional means of high copy-number species:

$$\mathbf{m}_z(\mathbf{y}, t) = \mathbb{E}_z[\mathbf{Z}|\mathbf{y}, t] = \sum_{\mathbf{z}\geq\mathbf{0}} \mathbf{z}p(\mathbf{z}|\mathbf{y}, t)$$

- Higher-order central conditional moments of high copy-number species

$$\mathbf{C}_{\mathbf{I},z}(\mathbf{y}, t) = \mathbb{E}_z\left[\big(\mathbf{Z} - \mathbf{m}_z(\mathbf{y}, t)\big)^{\mathbf{I}}|\mathbf{y}, t\right] = \sum_{\mathbf{z}\geq\mathbf{0}} \big(\mathbf{z} - \mathbf{m}_z(\mathbf{y}, t)\big)^{\mathbf{I}} p(\mathbf{z}|\mathbf{y}, t)$$

The evolution equations for marginal probabilities and conditional moments are derived from the CME, and

form a system of differential algebraic equations (DAEs):

$$
\begin{aligned}
\frac{\partial}{\partial t}p(\mathbf{y}|t) = & \sum_{j=1}^{n_r}\mathbb{E}_z[a_j(\mathbf{y}-\nu_{jy},\mathbf{Z})|\mathbf{y}-\nu_{jy},t]p(\mathbf{y}-\nu_{jy}|t) \\
& -\sum_{j=1}^{n_r}\mathbb{E}_z[a_j(\mathbf{y},\mathbf{Z})|\mathbf{y},t]p(\mathbf{y}|t), \\
p(\mathbf{y}|t)\frac{\partial}{\partial t}m_{i,z}(\mathbf{y},t) = & \sum_{j=1}^{n_r}\mathbb{E}_z[a_j(\mathbf{y}-\nu_{jy},\mathbf{Z})(Z_i-m_{i,z}(\mathbf{y}-\nu_{jy},t))|\mathbf{y}-\nu_{jy},t]p(\mathbf{y}-\nu_{j,y}|t) \\
& +\sum_{j=1}^{n_r}(m_{i,z}(\mathbf{y}-\nu_{jy},t)+\nu_{ij,z})\,\mathbb{E}_z[a_j(\mathbf{y}-\nu_{jy},\mathbf{Z})|\mathbf{y}-\nu_{j,y},t]p(\mathbf{y}-\nu_{j,y}|t) \\
& -\sum_{j=1}^{n_r}\left(\mathbb{E}_z[a_j(\mathbf{y},\mathbf{Z})(Z_i-m_{i,z}(\mathbf{y},t))|\mathbf{y},t]+m_{i,z}(\mathbf{y},t)\mathbb{E}_z[a_j(\mathbf{y},\mathbf{Z})|\mathbf{y},t]\right)p(\mathbf{y}|t) \\
& -m_{i,z}(\mathbf{y},t)\frac{\partial}{\partial t}p(\mathbf{y}|t), \\
p(\mathbf{y}|t)\frac{\partial}{\partial t}\mathbf{C}_{I,z}(\mathbf{y}|t) = & \sum_{j=1}^{n_r}\left(\sum_{0\le k\le I}\binom{I}{k}(\mathbf{m}_z(\mathbf{y}-\nu_{jy},t)-\mathbf{m}_z(\mathbf{y}|t)+\nu_{jz})^{I-k}\right. \\
& \left.\mathbb{E}_z[a_j(\mathbf{y}-\nu_{jy},\mathbf{Z})(\mathbf{Z}-\mathbf{m}_z(\mathbf{y}-\nu_{jy},t))^k|\mathbf{y}-\nu_{jy},t]p(\mathbf{y}-\nu_{jy}|t)\right) \\
& - \sum_{j=1}^{n_r}\mathbb{E}_z[a_j(\mathbf{y},\mathbf{Z})(\mathbf{Z}-\mathbf{m}_z(\mathbf{y},t))^I|\mathbf{y},t]p(\mathbf{y}|t) \\
& - \sum_{\substack{i=1\\ I_i\ge 1}}^{n_{s,z}} I_i\mathbf{C}_{I-e_i,z}(\mathbf{y},t)p(\mathbf{y}|t)\frac{\partial}{\partial t}m_{i,z}(\mathbf{y},t)-\mathbf{C}_{I,z}(\mathbf{y},t)\frac{\partial}{\partial t}p(\mathbf{y}|t).
\end{aligned}
\tag{1.10}
$$

Similar to moment equations, the evolution of lower-order conditional moments can depend on higher-order conditional moments, rendering moment closure necessary. The moment closure techniques introduced in Section 1.3.5 can be used to approximate the higher-order central conditional moments of the high copy-number species. The conditional moments and marginal probabilities can be used to calculate the overall moments as below:

- Mean of low copy-number species:

$$\bar{m}_{i,y}(t) = \sum_{\mathbf{y}\ge 0} y_i p(\mathbf{y}|t)$$

- Mean of high copy-number species:

$$\bar{m}_{i,z}(t) = \sum_{\mathbf{y}\ge 0} m_{i,z}(\mathbf{y}|t)p(\mathbf{y}|t)$$

- Higher-order central moments:

$$\bar{\mathbf{C}}_{\mathbf{I}}(t) = \sum_{y\ge 0}(\mathbf{y}-\bar{\mathbf{m}}_y(t))^{\mathbf{I}_y}\sum_{\mathbf{0}\le\mathbf{k}\le\mathbf{I}_z}\binom{\mathbf{I}_z}{\mathbf{k}}(\mathbf{m}_z(\mathbf{y}|t)-\bar{\mathbf{m}}_z(t))^{\mathbf{I}_z-\mathbf{k}}\mathbf{C}_{\mathbf{k},z}(\mathbf{y},t)p(\mathbf{y}|t)$$

Here $\mathbf{I}=(\mathbf{I}_y,\mathbf{I}_z)$, $\bar{\mathbf{m}}_y(t)=\big(\bar{m}_{1,y}(t),\ldots,\bar{m}_{n_{s,y},y}(t)\big)$, and $\bar{\mathbf{m}}_z(t)=\big(\bar{m}_{1,z}(t),\ldots,\bar{m}_{n_{s,z},z}(t)\big)$.

This hybrid description can yield an improved approximation accuracy [14].

1.4 Sensitivity analysis

The FSP, RRE, SSE, MM and MCM yield systems of differential equations. The parameters of differential equations can efficiently be inferred using gradient-based optimization methods [15]. While gradients can be approximated using finite differences, methods based on sensitivity equations are known to be more robust and computationally more efficient [15]. Sensitivity equations describe how much an aspect/functional of the process changes in response to a change in parameter values. CERENA implements forward and adjoint sensitivity analyses [16] for FSP, RRE, SSE, MM, and forward sensitivity analysis for MCM.

1.4.1 Forward sensitivity equations

Forward sensitivity equations provide the time-dependent sensitivity of the state-variables of the differential equations with respect to the parameters. Consider an n-dimensional ODE system

$$\begin{aligned} \dot{\mathbf{x}} &= f(\mathbf{x}, \theta, t), \quad \mathbf{x}(t_0) = \mathbf{x}_0(\theta) \\ \mathbf{y} &= h(\mathbf{x}, \theta, t), \end{aligned} \tag{1.11}$$

or an n-dimensional DAE system

$$\begin{aligned} &F(\dot{\mathbf{x}}, \mathbf{x}, \theta, t) = 0, \quad \mathbf{x}(t_0) = \mathbf{x}_0(\theta), \quad \dot{\mathbf{x}}(t_0) = \dot{\mathbf{x}}_0(\theta) \\ &\mathbf{y} = h(\mathbf{x}, \theta, t), \end{aligned} \tag{1.12}$$

where $\mathbf{x} \in \mathbb{R}^n$ denotes the state variables of the ODE/DAE system, $\mathbf{y} \in \mathbb{R}^{n_o}$ denotes the vector of output variables, and $\theta \in \mathbb{R}^{n_\theta}$ denotes the set of parameters. The forward sensitivities for these systems are defined as

$$\begin{aligned} \mathbf{S}^x(t) &= \left(\mathbf{s}_1^x(t), \mathbf{s}_2^x(t), \cdots, \mathbf{s}_{n_\theta}^x(t)\right) \in \mathbb{R}^{n \times n_\theta}, \\ \mathbf{s}_i^x(t) &= \frac{\partial \mathbf{x}(t)}{\partial \theta_i}, \quad \text{for } i = 1, 2, \cdots, n_\theta, \end{aligned} \tag{1.13}$$

where $\mathbf{s}_i^x(t) \in \mathbb{R}^n$ denotes the sensitivity of the state variables $\mathbf{x}$ with respect to the ith parameter, θ_i.

In the case of the ODE system, the forward sensitivity equations are

$$\begin{aligned} \dot{\mathbf{s}}_i &= \frac{\partial f}{\partial x}\mathbf{s}_i + \frac{\partial f}{\partial \theta_i}, \quad \text{for } i = 1, \cdots, n_\theta, \\ \mathbf{s}_i(t_0) &= \frac{\partial \mathbf{x}_0(\theta)}{\partial \theta_i}. \end{aligned} \tag{1.14}$$

In the case of a DAE system, the forward sensitivity equations are

$$\begin{aligned} &\frac{\partial F}{\partial x}\mathbf{s}_i + \frac{\partial F}{\partial \dot{x}}\dot{\mathbf{s}}_i + \frac{\partial F}{\partial \theta_i} = 0, \quad \text{for } i = 1, \cdots, n_\theta, \\ &\mathbf{s}_i(t_0) = \frac{\partial \mathbf{x}_0(\theta)}{\partial \theta_i}, \quad \dot{\mathbf{s}}_i(t_0) = \frac{\partial \dot{\mathbf{x}}_0(\theta)}{\partial \theta_i}. \end{aligned} \tag{1.15}$$

Assuming that the model possesses n state-variables and n_θ parameters, a system of $n(1 + n_\theta)$ differential equations is solved to compute the state sensitivities with respect to all parameters.

The sensitivity of output variables $\mathbf{y}$ is defined as

$$\begin{aligned} \mathbf{S}^y(t) &= \left(\mathbf{s}_1^y(t), \mathbf{s}_2^y(t), \cdots, \mathbf{s}_{n_\theta}^y(t)\right) \in \mathbb{R}^{n_o \times n_\theta}, \\ \mathbf{s}_i^y(t) &= \frac{\partial \mathbf{y}(t)}{\partial \theta_i}, \quad \text{for } i = 1, 2, \cdots, n_\theta, \end{aligned} \tag{1.16}$$

where $\mathbf{s}_i^y(t) \in \mathbb{R}^{n_o}$ denotes the sensitivity of the output variables $\mathbf{y}$ with respect to the ith parameter, θ_i. Having solved the forward sensitivity equations, the output sensitivities can be computed via

$$\mathbf{s}_i^y = \frac{\partial h}{\partial x}\,\mathbf{s}_i^x + \frac{\partial h}{\partial \theta_i}, \tag{1.17}$$

where $\frac{\partial h}{\partial x} = \left(\frac{\partial h_j}{\partial x_k}\right)_{jk} \in \mathbb{R}^{n_o \times n}$.

1.4.2 Adjoint sensitivity equations

If the sensitivity of few output functions with respect to many parameters is required, computing the state sensitivities is unnecessarily demanding, and solving adjoint sensitivity equations is more practical [16]. In adjoint sensitivity analysis, an adjoint state of size n is defined which is independent of the parameters. The adjoint state is obtained by solving the adjoint ODE system backward in time. The adjoint state can then be used to calculate the sensitivity with respect to any parameter of interest by computing a one-dimensional integral. Therefore, the adjoint sensitivity of each output function with respect to n_θ parameters roughly requires forward integration of a system of n ODEs, backward integration of a system of n ODEs and calculating n_θ one-dimensional integrals. Thus, in applications with high-dimensional parameter spaces and/or few output functions, calculating adjoint sensitivities tends to be computationally more advantageous. In parameter estimation, the likelihood function can be defined as the sole output of the system, which enables efficient exploitation of adjoint sensitivity analysis.

1.5 Likelihood function evaluation

CERENA can be linked to optimization toolboxes to perform parameter estimation. To facilitate this procedure, CERENA offers the evaluation of the likelihood of the user-provided data under the assumption that the noise is normally distributed. If we denote the model prediction for the observables by $\mathbf{y}$, the negative log-likelihood function evaluated by CERENA is written as:

$$J(\theta) = \frac{1}{2}\sum_{k,i}\left(\log 2\pi + \log \sigma_{i,k}^2 + \frac{\left(y_i(t_k,\theta) - \hat{y}_{i,k}\right)^2}{\sigma_{i,k}^2}\right), \tag{1.18}$$

where $\hat{y}_{i,k}$ denotes the measurement data for the ith observable at time point t_k, and $\sigma_{i,k}$ denotes the standard deviation of the normally-distributed noise. The maximum likelihood estimate for the parameters is given by

$$\hat{\theta} = \arg\min_{\theta \in \Theta} J(\theta), \tag{1.19}$$

with Θ being a plausible search domain for parameters.

The negative log-likelihood value $J(\theta)$, returned by CERENA, can be passed to minimization tools for parameter estimation.

Chapter 2

CERENA startup

2.1 Installation

To install the CERENA toolbox, go to http://cerenadevelopers.github.io/CERENA/ and download the `.tar` file. Copy the downloaded tar file to the desired installation path and extract it using the following command:

```
tar xvf filename.tar
```

filename is the name of the `.tar` file.

CERENA folder contains the following directories:

- `CERENA/lib`: Includes all the scripts of CERENA, structured as follows:
  - `CERENA/lib/compilation_tools` contains the scripts for generating MATLAB-based and SUNDIALS-based simulation files.
  - `CERENA/lib/moment_equations` contains the scripts for the derivation of moment equations and conditional moment equations.
  - `CERENA/lib/system_size_expansion` contains the scripts for the derivation of system size expansion equations.
  - `CERENA/lib/CME_tools` contains the scripts for the finite state projection and stochastic simulation algorithms.
  - `CERENA/lib/auxiliary` contains the auxiliary scripts used in other routines.
  - `CERENA/lib/visualization_tools` contains the visualization routines.
- `CERENA/examples`: Includes biological systems implemented in CERENA. The three-stage gene expression example used throughout this documentation can also be found in `CERENA/examples/geneExpression`.
- `CERENA/doc`: Includes the CERENA documentation.

To use CERENA, `install_cerena.m` must be run at the beginning of each MATLAB session. This step adds the CERENA path to the top of MATLAB search path. Alternatively, the CERENA path can be permanently added to MATLAB search path, in which case the execution of `install_cerena.m` in the beginning of each session will not be necessary.

2.2 Requirements

CERENA is installed and tested on Mac OS X 10.9 and 10.10 and Linux. However, configuration of the toolbox on Windows is yet to be completed. Therefore, depending on the Windows version, users might experience difficulties compiling the simulation files. While we keep working on this issue, users are encouraged to send us their feedback on configuring CERENA.

CERENA requires the installation of following software/toolboxes:

- **MATLAB.** CERENA is implemented in MATLAB, and therefore all functionalities of CERENA require MATLAB software.
- **MATLAB Symbolic Math Toolbox.** CERENA extensively uses MATLAB Symbolic Math Toolbox for the derivation of equations and the compilation of simulation files.
- **SUNDIALS CVODES and IDAS packages.** The compilation of simulation MEX-files in CERENA relies on CVODES and IDAS packages. These packages are included in the corresponding wrappers, i.e. `CERENA/compilation_tools/cvodewrap` and `CERENA/compilation_tools/idawrap`. Therefore, the user does **not** need to install CVODES and IDAS packages separately.
- **SBML Toolbox.** The usage of `importSBML` (see Section 3.2) requires the installation of SBML Toolbox [17].

Chapter 3

Setting up a model in CERENA

To simulate a chemical reaction network in CERENA, first the biochemical reaction network has to be specified. The network specification is provided by the user in the form of a *model definition file.* Alternatively, networks specified in the Systems Biology Markup Language (SBML) format can be imported.

3.1 Model definition file

A model definition file is an m-file that contains all the necessary specifications of the biochemical reaction network. The model definition file will be referred to as `modelDef.m` in this documentation. `modelDef.m` assembles a `System` structure array with the following fields:

- `System.time` is the symbolic variable used to denote time. This symbolic variable is required in handling of time-dependent expressions, e.g., in input functions or reaction propensities.
- `System.compartments` is a string cell array containing the names of the compartments of the chemical reaction network.

```
System.compartments = {'comp_1';'comp_2'; ···}
```

- `System.volumes` is a symbolic or numeric array containing the compartment volumes in the same order as `System.compartments`.

```
System.volumes = [vol_1; vol_2; ···]
```

- `System.state` is a field containing the following information about the chemical species or the *states* of the system:
  - `System.state.variable` is an array of symbolic variables denoting the chemical species.

```
System.state.variable = [x_1; x_2; x_3; ···]
```

  - `System.state.compartment` is an array specifying the compartment to which each species belongs. This array is defined in the same order as `System.state.variable`.

```
System.state.compartment = {'comp_1'; 'comp_1'; 'comp_2'; ···}
```

– `System.state.name` (optional) is a string cell array containing the names of species in the same order as `System.state.variable`. By default, the names will be set to the string version of `System.state.variable`.

```
System.state.name = {'species_1'; 'species_2'; 'species_3'; ···}
```

– `System.state.type` (optional) is a string cell array in the order of `System.state.variable` that determines whether a microscopic or a mesoscopic description for each species should be used (see Section 1.3.6). This field is only necessary for MCM simulations. For microscopic descriptions, `stochastic` type and for mesoscopic descriptions `moment` type should be specified. If unspecified, all types will be set to `moment` by default.

```
System.state.type = {'stochastic'; 'moment'; 'moment'; ···}
```

– `System.state.mu0` is a symbolic or numeric vector of the initial values of the mean of species in the same order as `System.state.variable`.

`System.state.mu0 = [`m_{0_1}`;` m_{0_2}`;` m_{0_3}`; ···]`

Attention! The initial conditions should be provided in *molecule numbers* and not in concentrations.

– `System.state.C0` is a symbolic or numeric vector of the initial values of the covariances of species. If unspecified, all covariances will be set to zero by default.

`System.state.C0 = [`$C_{0_{1,1}}$`;` $C_{0_{1,2}}$`;` $C_{0_{1,3}}$`; ···]`

Attention! The ordering of the covariances in `System.state.C0` should be

$$[C_{1,1}, C_{1,2}, \cdots, C_{1,n_s}, C_{2,2}, \cdots, C_{2,n_s}, \cdots, C_{n_s-1,n_s-1}, C_{n_s-1,n_s}, C_{n_s,n_s}].$$

Attention! The initial conditions should be provided in (*molecule number*)^2 and not in (concentration)^2.

Attention! The initial conditions specified here for `mu0` and `C0` can be overwritten before and after the compilation of simulation files. See Section 5.1 for more details.

– The following fields are only required for FSP and MCM simulations, and are used to construct the state space of FSP or MCM:

  ∗ `System.state.xmin` specifies the lower bound on the count of species.

```
System.state.xmin = [x_min_1; x_min_2; x_min_3; ···]
```

  ∗ `System.state.xmax` specifies the upper bound on the count of species.

```
System.state.xmax = [x_max_1; x_max_2; x_max_3; ···]
```

  ∗ `System.state.constraint` is a function that defines any constraints that should be imposed on the state of the system. The output of this function should be a logical value (true or false).

```
System.state.constraint = @(x) f(x)
```

– The specified lower and upper bounds and the constraint are used to construct the state space of FSP/MCM simulations. More specifically, the state space of FSP is defined as:

$$\forall \mathbf{x} \in \mathbb{N}_0^{n_s} : \mathbf{x}_{\min} \leq \mathbf{x} \leq \mathbf{x}_{\max} \ \wedge \ f(x) \tag{3.1}$$

Similarly, the regime of the low copy-number species in the MCM simulations are defined in this way (see Section 1.3.6).

- `System.parameter` contains the following information about the parameters of the system, e.g., kinetic rates.

Attention! The sensitivity equations are derived for all parameters defined in `System.parameter`. To reduce the compilation time, the parameters for which the sensitivities are not required can be defined in `System.kappa` (see below).

  – `System.parameter.variable` is a symbolic array including the variables that denote the parameters of the system, e.g., the kinetic rates.

```
System.parameter.variable = [p_1; p_2; p_3; ···]
```

  – `System.parameter.name` (optional) is a string array of the parameter names in the same order as `System.parameter.variable`. By default, the parameter names are set to the string version of `System.parameter.variable`.

```
System.parameter.name = {'par_1'; 'par_2'; 'par_3'; ···}
```

- `System.kappa` contains the following information about the constant parameters of the system, i.e., the parameters for which the derivation of sensitivity equations is not required.

  – `System.kappa.variable` is a symbolic array including the variables that denote the constant parameters of the system.

```
System.kappa.variable = [k_1; k_2; k_3; ···]
```

  – `System.kappa.name` (optional) is a string array of the names of the constant parameters in the same order as `System.kappa.variable`. By default, the names are set to the string version of `System.kappa.variable`.

```
System.kappa.name = {'kappa_1'; 'kappa_2'; 'kappa_3'; ···}
```

- `System.reaction` is a field specifying the chemical reactions of the system, containing the following subfields:

  – `System.reaction(j).educt` is a symbolic vector of the reactants of the jth reaction. Each species, S_i, should be repeated $\nu_{i,j}^-$ times where $\nu_{i,j}^-$ is the reactant stoichiometric coefficient of S_i in the jth reaction.

```
System.reaction(j).educt = [x_1, ···]
```

  – `System.reaction(j).product` is a symbolic vector of the products of the jth reaction. Each species, S_i, should be repeated $\nu_{i,j}^+$ times where $\nu_{i,j}^+$ is the product stoichiometric coefficient of S_i in the jth reaction.

```
System.reaction(j).product = [x_2, ···]
```

– `System.reaction(j).propensity` is the symbolic expression of the propensity of the jth reaction. The propensities generally depend on the abundance of species, $\mathbf{x}$, and the parameters $\mathbf{p}$ and $\mathbf{k}$.

```
System.reaction(j).propensity = g(p,k,x)
```

Attention! Some of the modeling approaches implemented in CERENA require the microscopic propensities and some require the macroscopic rate functions. However, the user should only specify either of the two, and specify the scale using `System.scaleIndicator` (see below). If a conversion from one scale to the other is necessary for the selected modeling approach, CERENA will automatically carry out the scale conversion. More information on the definition of microscopic and macroscopic rate functions, and the conversion of the two into each other can be found in Section 1.1.

- `System.scaleIndicator` specifies the scale of the propensities and parameter. This indicator should be set to `'microscopic'` if the microscopic propensities and parameters are provided, and should be set to `'macroscopic'` if macroscopic rate functions and parameters are provided (see Section 1.1).

```
System.scaleIndicator = 'microscopic'   or   System.scaleIndicator = 'macroscopic'
```

- `System.output` is a field that includes the information about the observables of the system, or any functions of the states of the system.

  – `System.output.variable` is an array of the symbolic variables used to denote the output variables.

```
System.output.variable = [y_1; y_2; ···]
```

  – `System.output.name` (optional) is a string array containing the names of the outputs in the same order as `System.output.variable`. By default, the names will be set to the string version of `System.output.variable`.

```
System.output.name = {'observable_1'; 'observable_2'; ···}
```

  – `System.output.function` is an array containing the symbolic expressions of the output functions that correspond to the output variables. The output functions can depend on the abundance of species, $\mathbf{x}$, and the parameters $\mathbf{p}$ and $\mathbf{k}$.

```
System.output.function = [fy_1(p,k,x); fy_2(p,k,x); ···]
```

- `System.input` is a field that includes the information about the input signals to the chemical reaction network such as stimuli.

  – `System.input.variable` is an array of the symbolic variables used to denote the input variables.

```
System.input.variable = [u_1; ···]
```

  – `System.input.name` (optional) is a string array containing the names of the inputs in the same order as `System.input.variable`. By default, the names will be set to the string version of `System.input.variable`.

```
System.input.name = {'stimulus_1'; ···}
```

– System.input.function is an array containing the symbolic expressions of the input functions that correspond to the input variables. The input functions can depend on time.

```
System.input.function = [fu_1(t); ···]
```

The unspecified fields and default settings will then be added to the System structure array using completeSystem.m function. This step is automatically done in CERENA as explained in Chapter 4. The System structure defined in this way will have all the necessary information for simulating the reaction network in CERENA.

3.1.1 Example: The three-stage gene expression model

The modelDef.m file for the three-stage gene expression example is shown below. This file (modelDef_geneExpression.m) can be found in CERENA/examples/geneExpression folder.

```
%% MODEL DEFINITION
% Definition of symbolic variables:
syms DNA_off DNA_on mRNA Protein %  species
syms scaledProtein % observables
syms tau_on tau_off k_m gamma_m k_p gamma_p tau_on_p % kinetic rates
syms Omega % volume
syms scaleP offsetP % scaling parameters for the observable
syms r0 % initial count of mRNA
syms time % time

% Definition of general fields:
System.time = time;
System.compartments = {'cell'};  % The name of the compartment, e.g., cytoplasm
System.volumes = [Omega];      % The volume of the compartment

% Definition of state field:
System.state.variable       = [DNA_off; DNA_on;  mRNA; Protein];
System.state.compartment    = { 'cell'; 'cell';'cell';  'cell'};
System.state.name           = {'DNA_{off}' ; 'DNA_{on}' ;  'mRNA';'Protein'};
System.state.type           = {'stochastic';'stochastic';'moment'; 'moment'};
System.state.xmin           = [      0     ;      0     ;   0   ;    0   ];
System.state.xmax           = [      1     ;      1     ;   40  ;   150  ];
System.state.mu0            = [      1     ;      0     ;   r0  ;     0  ];
System.state.C0             = zeros(System.state.number*(System.state.number+1)/2,1);
System.state.constraint     = @(x) ((x(1)+x(2)) == 1);

% Definition of parameters field:
System.parameter.variable   = [ tau_on; tau_off; k_m ; gamma_m; k_p ; gamma_p; tau_on_p;
                                scaleP; offsetP; r0];
System.parameter.name   = {'\\tau_{on}'; '\\tau_{off}'; 'k_m'; '\\gamma_m'; 'k_p'; '\\gamma_p';
                           '\\tau_{on,p}'; 'scale_p'; 'offset_p'; 'r_0'};

% Definition of constant parameters field:
System.kappa.variable = [Omega];

% Specifying the scale of propensities and parameters
System.scaleIndicator = 'microscopic';  % options are 'microscopic' and 'macroscopic'

% Definition of reactions:
% (R1)
System.reaction(1).educt        = DNA_off;
```

```
System.reaction(1).product      = DNA_on;
System.reaction(1).propensity   = tau_on*DNA_off;
% (R2)
System.reaction(2).educt        = DNA_on;
System.reaction(2).product      = DNA_off;
System.reaction(2).propensity   = tau_off*DNA_on;
% (R3)
System.reaction(3).educt        = DNA_on;
System.reaction(3).product      = [mRNA,DNA_on];
System.reaction(3).propensity   = k_m*DNA_on;
% (R4)
System.reaction(4).educt        = mRNA;
System.reaction(4).product      = [];
System.reaction(4).propensity   = gamma_m*mRNA;
% (R5)
System.reaction(5).educt        = mRNA;
System.reaction(5).product      = [Protein,mRNA];
System.reaction(5).propensity   = k_p*mRNA;
% (R6)
System.reaction(6).educt        = Protein;
System.reaction(6).product      = [];
System.reaction(6).propensity   = gamma_p*Protein;
% (R7)
System.reaction(7).educt        = [DNA_off,Protein];
System.reaction(7).product      = [DNA_on ,Protein];
System.reaction(7).propensity   = tau_on_p*Protein*DNA_off;

System.output.variable  = [scaledProtein ];
System.output.name      = {'scaled_Protein'};
System.output.function  = [offsetP + scaleP * Protein];
```

3.2 SBML Import

If the SBML specification of the reaction network is available, it is not necessary to manually create a `modelDef.m` file. Instead, CERENA creates the `modelDef.m` by using an automatic SBML import, `importSBML.m`:

```
System = importSBML('model_name')
```

where `model_name` (string) is the name of the corresponding SBML file. The generated `System` structure array can be altered in MATLAB by overwriting individual fields. In particular, the user is encouraged to make sure about the correct scale of the propensities in the generated `modelDef.m` file.

Attention! By default, `importSBML.m` adds all the parameters to `System.parameter` field, and leaves the `System.kappa` field empty. The user can change this by editing the generated `modelDef.m` file.

3.2.1 Example: The three-stage gene expression model

The three-stage gene expression model has been specified in SBML, and stored in `CERENA/examples/SBML_example/GeneExpressionCopasi.xml`.

The following command automatically generates a `modelDef.m` file for this example, named `modelDef_GeneExpressionCopasi.m`:

```
System = importSBML('GeneExpressionCopasi')
```

It also returns the `System` structure array as the output. The `modelDef.m` generated in this way can be found in `CERENA/examples/SBML_example`.

Chapter 4

Compiling Simulation Files

In this chapter, we demonstrate how CERENA can be used to derive the governing equations for the method of interest, and to compile simulation MEX-files.

4.1 Generation of simulation files

After creating the `modelDef.m` file, one of the following commands can be used to generate the system of equations and compile simulation files:

- For generating the C code for the simulation of ODE systems, e.g., FSP, RRE, SSE, and MM, the `genSimFile` command is used:

```
System = genSimFile(modelName,modelDefName,method)
```

- For generating the C code for the simulation of ODE systems, e.g., FSP, RRE, SSE, and MM, together with the evaluation of a likelihood function (see Section 1.5), the `genSimFileLLH` command is used:

```
System = genSimFileLLH(modelName,modelDefName,method)
```

- For generating the C code for the simulation of DAE systems, e.g., MCM, the `genSimFileIDA` command is used:

```
System = genSimFileIDA(modelName,modelDefName,method)
```

The input arguments are:

- `modelName` is a string providing the name of the model. This name will be used in naming the simulation files.
- `modelDefName` is the name of the `modelDef.m` file.
- `method` is the selected modeling approach. The possible options for `method` are presented in Section 4.2.

The output argument is the `System` structure array defined earlier. Running these commands will add model-specific fields to `System` which contain information about the selected modeling approach.

The three commands above consist of three main functions: `completeSystem()`, `genmexp()` and one of the wrappers for SUNDIALS solvers (`cvodewrap()`, `idaswrap()`, and `llhwrap()`).

completeSystem()

This function is used to add the unspecified and default fields to the `System` structure array. Most importantly, the interconversion of microscopic and macroscopic rate functions is performed in this step.

```
System = completeSystem(System)
```

genmexp()

This function is used to derive the system of governing equations for the selected modeling approach. Executing this command also creates an m-file named `method_modelName_syms`. This file contains the symbolic representation of the system, including the equations and corresponding initial conditions.

Additionally, an m-file is created for the numerical simulation of the system using MATLAB `ode15s` solver, named `method_modelName_matlab`. The generation of this intermediate simulation file is optional, and is meant for simplicity in cases where the user wishes to perform numerical simulations using MATLAB. However, the simulation MEX-files generated by CERENA do not rely on this m-file.

```
System = genmexp(modelName,modelDefName,method)
```

Here, the input and output arguments are the same as those in `genSimFile()`.

cvodewrap()

In case the governing equations are a system of ODEs, this function is used to compile the MEX-file used for numerical simulation, named `modelName`. The compilation of this MEX-file relies on the SUNDIALS CVODES package, and therefore the corresponding CVODES options can be specified as inputs (see Section 5.1 for more details). Also, a MATLAB interface for calling this MEX-file with the appropriate options and input arguments is generated. This m-file, named `simulate_modelName`, should be called to run the numerical simulation using MEX-files (see Section 5.1).

```
cvodewrap(modelName,method_modelName_syms)
```

`method_modelName_syms` is the name of the m-file containing the symbolic representation of the governing equations.

llhwrap()

In case the governing equations are a system of ODEs, and the user wishes to calculate an objective function with additive normally distributed measurement noise (see Section 1.5) `llhwrap` can be used to compile the corresponding MEX-file, named `modelName`. The compilation of this MEX-file relies on the SUNDIALS CVODES package, and therefore the corresponding CVODES options can be specified as inputs (see Section 5.1 for more details). Also, a MATLAB interface for calling this MEX-file with the appropriate options and input arguments is generated. This m-file, named `llh_modelName`, should be called to run the numerical simulation using MEX-files (see Section 5.1).

```
llhwrap(modelName,method_modelName_syms)
```

`method_modelName_syms` is the name of the m-file containing the symbolic representation of the governing equations.

idawrap()

In case the governing equations are a system of DAEs, this function is used to compile the MEX-file used for numerical simulation, named `modelName`. The compilation of this MEX-file relies on the SUNDIALS IDAS package, and therefore the corresponding IDAS options can be specified as inputs (see Section 5.1 for more details). Also, a MATLAB interface for calling this MEX-file with the appropriate options and input arguments is generated. This m-file, named `simulate_modelName`, should be called to run the numerical simulation using MEX-files (see Section 5.1).

```
idawrap(modelName,method_modelName_syms)
```

`method_modelName_syms` is the name of the m-file containing the symbolic representation of the governing equations.

All the above three steps are contained in `genSimFile`, `genSimFileLLH` and `genSimFileIDA`, and therefore the user only needs to call one of these functions to generate all the necessary simulation files.

4.2 Specification of modeling approach

The possible options for modeling approaches include different orders of SSE (Section 1.3.4), different orders of MM (Section 1.3.5), different orders of MCM (Section 1.3.6), and FSP (Section 1.3.2).

4.2.1 System Size Expansion

To specify different orders of SSE as the modeling approach, the `method` is set to the following values:

Modeling Approach	`method`
SSE 1st-order (RRE)	`'RRE'`
SSE 2nd-order (LNA)	`'LNA'`
SSE 3rd-order (EMRE)	`'EMRE'`
SSE 4th-order (IOS)	`'IOS'`

The system of SSE ODEs are then derived for the state variables. In addition, equations for the calculation of the moments of species based on the state variables are derived. Also, the equations for the mean and the variance* of the output variables are derived and presented in the `method_modelName_syms` file. The initial conditions `System.state.mu0` provided in the `modelDef.m` file are used as the initial conditions for the mean of species. The initial conditions for the rest of the state variables are set to zero.

Attention! The SSE equations can only be derived for the concentration of species. However, the initial conditions in `modelDef.m` should be given in molecule numbers. The conversion of the initial conditions to concentrations is automatically done when generating SSE equations.

Attention! These initial conditions can be overwritten before the compilation of MEX-files, or for the numerical simulation. See Sections 4.3.1 and 5.1 for instructions.

*In case of RRE only the equations for the mean of the output variables is derived.

4.2.2 Method of Moments

To specify different orders of MM as the modeling approach, the `method` should be given in the following form:

```
'MEC_XO_MC_YO_SC'
```

where

- `MEC` stands for the "central moment equations", and should be kept unchanged.
- `XO` specifies the truncation order for the moment equations of the state variables.
- `MC` specifies the moment closure scheme (see Section 1.3.5). Any of the following options can be chosen for the closure scheme:
  - `LD` for low dispersion closure technique.
  - `ZC` for zero cumulants closure technique.
  - `MF` for mean field closure technique.
  - `DM` for derivative matching closure technique.
  - `UD` for user-defined closure technique (see Section 4.5).
- `YO` specifies the order of the output moments, and can be smaller than or equal to the truncation order `XO`. This value does not change the truncation order of the moment equations and merely determines which moments of the output variables should be calculated based on the moments of the species.
- `SC` specifies the scale of the equations which can be either of the following:
  - `a` specifies that the moment equations for the molecule numbers of species should be derived.
  - `c` specifies that the moment equations for the concentration of species should be derived.

The system of ODEs are derived for the first `XO` moments of the species. In addition, equations for the calculation of the first `YO` moments of the outputs are derived and presented in the `MEC_XO_MC_YO_SC_modelName_syms` file. The initial conditions `System.state.mu0` provided in the `modelDef.m` file are used as the initial conditions for the mean of species. The initial conditions `System.state.C0` provided in the `modelDef.m` file are used as the initial conditions for the (co)variances of species. The initial conditions for the rest of the state variables are set to zero.

Attention! Regardless of the scale of the moment equations `SC`, the initial conditions should be provided in molecule numbers. If necessary, the conversion to concentration scale is automatically performed when generating MM equations.

Attention! These initial conditions can be overwritten before the compilation of MEX-files, or for the numerical simulation. See Sections 4.3.1 and 5.1 for instructions.

4.2.3 Method of Conditional Moments

To specify different orders of MCM as the modeling approach, the `method` should be given in the following form:

```
'CMEC_XO_MC_YO_SC'
```

where

- `CMEC` stands for the "central conditional moment equations", and should be kept unchanged.

- `XO` specifies the truncation order for the conditional moment equations.
- `MC` specifies the moment closure scheme (see Section 1.3.6). Any of the following options can be chosen for the closure scheme:
  - `LD` for low dispersion closure technique.
  - `ZC` for zero cumulants closure technique.
  - `MF` for mean field closure technique.
  - `DM` for derivative matching closure technique.
  - `UD` for user-defined closure technique (see Section 4.5).
- `YO` specifies for the order of the output moments, and can be smaller than or equal to the truncation order `XO`. This value does not change the truncation order of the conditional moment equations and merely determines which moments of the output variables should be calculated based on the state variables.
- `SC` specifies the scale of the equations which can be either of the following:
  - `a` specifies that the conditional moment equations for the molecule numbers of species should be derived.
  - `c` specifies that the conditional moment equations for the concentration of species should be derived.

The system of DAEs are derived for the marginal probabilities of `'stochastic'` species and the first `XO` conditional moments of the `'moment'` species (see Section 1.3.6). In addition, equations for the calculation of the first `XO` overall moments of species, as well as the first `YO` moments of the outputs are derived and presented in the `CMEC_XO_MC_YO_SC_modelName_syms` file.

Attention! Regardless of whether the output functions include `'stochastic'` species, the outputs are always given in terms of their moments, ad no marginal probabilities for the outputs are calculated.

The initial conditions `System.state.mu0` provided in the `modelDef.m` file are used as the initial conditions for

- (i) the number of molecules for the `'stochastic'` species, and
- (ii) the conditional mean of the counts of the `'moment'` species.

Therefore, the *stochastic state* that corresponds to the initial molecule numbers of the `'stochastic'` species is given an initial marginal probability of 1. The rest of the *stochastic states* will have a zero initial marginal probability.

The initial conditions `System.state.C0` provided in the `modelDef.m` file are used as the initial conditions for the (co)variances of `'moment'` species conditioned on the *stochastic state* with initial probability of 1. The initial conditions for the rest of the state variables are set to zero.

Attention! Regardless of the scale of the conditional moment equations `SC`, the initial conditions should be provided in molecule numbers. If necessary, the conversion to concentration scale is automatically performed when generating MCM equations.

Attention! These initial conditions can be overwritten before the compilation of MEX-files, or for the numerical simulation. See Sections 4.3.1 and 5.1 for instructions.

4.2.4 Finite State Projection

To specify FSP as the modeling approach, the `method` is simply set to `'FSP'`. The state space of FSP is constructed by considering all combinations of the species counts that are

- in the interval [`System.state.xmin`, `System.state.xmax`], and
- satisfy the constraints defined by `System.state.constraint`.

The system of ODEs for the probabilities of the FSP state variables is derived. In addition, the equations for the calculation of the 1st and 2nd-order moments of the output variables are derived and presented in the `FSP_modelName_syms` file.

The initial conditions `System.state.mu0` provided in the `modelDef.m` file are used as the initial conditions for the initial molecule numbers of species. Therefore, the FSP state that corresponds to the provided initial molecule numbers is given an initial probability of 1. The rest of the states will have a zero initial probability. The initial conditions `System.state.C0` provided in the `modelDef.m` file are not used in FSP simulation.

Attention! The FSP equations can only be derived for the number of molecules of species.

Attention! These initial conditions can be overwritten before the compilation of MEX-files, or for the numerical simulation. See Sections 4.3.1 and 5.1 for instructions.

4.2.5 Symbolic representation

As explained in the beginning of this Chapter, an m-file, entitled `method_modelName_syms.m`, containing the symbolic representation of the derived system of equations, together with the initial conditions is generated by executing the following command:

```
genmexp(modelName,modelDefName,method)
```

This file is the basis for compiling the simulation MEX-files in later steps. The user can consult this file to easily access the equations and initial conditions.

4.2.6 Other formats

In addition to the aforementioned files, a model definition file in the format required by Data2Dynamics software [18] is generated.

4.3 Compilation of simulation MEX-files

CERENA uses wrappers for SUNDIALS CVODES and IDAS packages to compile MEX-files for the numerical simulation of the system and the sensitivity equations. In the case of a system of ODEs, either of the following commands compiles a MEX-file (`modelName`) and the corresponding MATLAB interface (`simulate_modelName.m` or `llh_modelName.m`) that can be called by the user (see Section 5.1):

- Simulation of the ODE system

```
cvodewrap(modelName,method_modelName_syms)
```

- Simulation of the ODE system and evaluation of a likelihood function (see Section 1.5)

```
llhwrap(modelName,method_modelName_syms)
```

Similarly, in the case of a system of DAEs, the following command

```
idawrap(modelName,method_modelName_syms)
```

compiles a MEX-file and the corresponding MATLAB interface that can be called by the user (see Section 5.1). Using these commands, the forward sensitivity equations (`cvodewrap`, `llhwrap` and `idawrap`) and adjoint sensitivity equations (`cvodewrap` and `llhwrap`) with respect to all the non-constant parameters are derived (see Section 3.1). However, when carrying out numerical simulations, the user can specify which sensitivity equations should be solved (see Section 5.4).

By default, the compiled simulation files will be stored in

`CERENA/lib/compilation_tools/wrap/models/modelname`, with `wrappername` being one of `cvodewrap`, `llhwrap` and `idawrap`. When using `cvodewrap`, a target directory for storing the compiled simulation files can be specified as below

```
cvodewrap(modelName,method_modelName_syms,tdir)
```

where `tdir` is the path of the target directory.

4.3.1 Rewriting the initial conditions before the compilation of MEX-files

Before compiling the MEX-files for the numerical simulation, the user can overwrite previously specified initial conditions in two different ways:

- The initial conditions in the symbolic representation file (`method_modelName_syms.m`) can be manually altered by the user.
- The user can provide a numeric or symbolic vector of initial conditions as an input to `cvodewrap`/`idawrap`, as below

  ```
  cvodewrap(modelName,method_modelName_syms,tdir,IC)
  ```

  ```
  idawrap(modelName,method_modelName_syms,IC)
  ```

  Here, `IC` is the vector of initial conditions in the same order as [`System.state.mu0`; `System.state.C0`].

4.4 Example: The three-stage gene expression model

In the following an excerpt of code to generate and compile the simulation files for the three-stage gene expression example using IOS is given:

```
modelDefName = 'modelDef_geneExpression'; % The name of the model definition file
modelName = 'geneExpressionIOS'; % The name that will be used for naming the simulation files
method = 'IOS'; % Specifying the modeling approach
System_IOS = genSimFile(modelName,modelDefName,method)
```

This will generate `IOS_geneExpressionIOS_syms.m` and `IOS_geneExpressionIOS_matlab.m` in the same directory as `modelDef_geneExpression.m` file.

The simulation files, i.e. `geneExpressionIOS` and `simulate_geneExpressionIOS` will be stored in `CERENA/lib/compilation_tools/cvodewrap/models/geneExpressionIOS`.

In order to compile simulation files using other ODE-based modeling approaches, only the line specifying the `method` needs to change. For example, to use the 3rd-order MM (`MEC_3`) with low dispersion closure (`_LD`) and 2nd-order outputs (`_2`) for the calculation of the concentration of species (`_c`), the following should be specified:

```
modelName = 'geneExpressionMM'; % The name that will be used for naming the simulation files
method = 'MEC_3_LD_2_c'; % Specifying the modeling approach
System_MM = genSimFile(modelName,modelDefName,method)
```

To use the 2nd-order MCM (`CMEC_2`) with zero cumulants closure (`_ZC`) and 2nd-order outputs (`_2`) for the calculation of the molecule numbers of species (`_a`), the following should be specified:

```
modelName = 'geneExpressionMCM'; % The name that will be used for naming the simulation files
method = 'CMEC_2_ZC_2_a'; % Specifying the modeling approach
System_MCM = genSimFileIDA(modelName,modelDefName,method)
```

Here, `genSimFileIDA` is used to generate the simulation files, as MCM yields a system of DAEs.

`genSimFileLLH` can be used to compile a simulation file which also evaluates the likelihood function value:

```
modelName = 'geneExpressionRRE'; % The name that will be used for naming the simulation files
method = 'RRE'; % Specifying the modeling approach
System_RRE = genSimFileLLH(modelName,modelDefName,method)
```

4.5 User-defined closure technique

In order to provide more degrees of freedom in the choice of moment closure technique, user-defined closures can be provided as a function, named `ud_closure.m`, with the following input and output arguments,

```
[hoM_closure,flag,moment_closure_rest] = ud_closure(hoM, order, System)
```

where

- `hoM` is an $n_1 \times n_2$ matrix containing the higher-order moments to be represented in terms of lower-order moments by the used-defined closure. Here, n_1 is the number of higher-order moments and n_2 is the order of the highest-order moment in `hoM`. Each row represents a higher-order moment where each element could either be zero or the index of a species (see Section 4.5.1).
- `order` is the truncation order of the moment equations.
- `System` is the `System` structure array.
- `hoM_closure` is a symbolic array of the length n_1, i.e. the number of higher-order moments, containing the symbolic representation of the corresponding higher-order moments in terms of lower-order moments. The symbolic variables used in `hoM_closure` should follow a specific form as explained below.
- `flag` (optional) if the user-determined moment closure provides substitutions only for certain higher-order moments, this output argument can be used to determine which higher-order moments have been

represented by the user-defined closure. `flag` is a numeric or logical array of length n_1, i.e. the number of higher-order moments, with 0 or 1 entries. A 1 (0) entry specifies that the corresponding higher-order moment has (not) been represented by the user-defined closure. The higher-order moments with a 0 flag will be substituted by one of the built-in closure techniques in CERENA.

- `moment_closure_rest` (optional) specifies which of the built-in closure techniques in CERENA should be used for the higher-order moments with the flag 0. This output argument can have one of these four values: `'low-dispersion'`, `'zero-cumulants'`, `'mean-field'` and `'derivative-matching'`. If this output argument is not provided, by default the `'zero-cumulants'` closure will be used.

Specific format of `hoM_closure`. `hoM_closure` returned by the `ud_closure` function, is a symbolic array containing symbolic expressions of higher-order moments in terms of lower-order moments. For compatibility of this variable with the rest of variables in CERENA, the symbolic variables representing the moments in `hoM_closure` should have the following format:

- The mean of the ith species is named `mu_i`.
- The central moment between the species i, j, k, $\cdots$, where $i \leq j \leq k \leq \cdots$, is named `C_i_j_k_`$\cdots$.

4.5.1 Example: The three-stage gene expression model

The excerpt of code below shows exemplary inputs and outputs of a user-defined closure function:

```
%  The vector of higher-order moments involved in
%  the third-order moment equations for the three-stage gene expression example
hoM =  [1     1     1     4;  % E[(x_1-mu_1)^3*(x_4-mu_4)]
        1     1     2     4;  % E[(x_1-mu_1)^2*(x_2-mu_2)*(x_4-mu_4)]
        1     1     3     4;  % E[(x_1-mu_1)^2*(x_3-mu_3)*(x_4-mu_4)]
        1     1     4     4;  % E[(x_1-mu_1)^2*(x_4-mu_4)^2]
        1     2     2     4;  % E[(x_1-mu_1)*(x_2-mu_2)^2*(x_4-mu_4)]
        1     2     3     4;  % E[(x_1-mu_1)*(x_2-mu_2)*(x_3-mu_3)*(x_4-mu_4)]
        1     2     4     4;  % E[(x_1-mu_1)*(x_2-mu_2)*(x_4-mu_4)^2]
        1     3     3     4;  % E[(x_1-mu_1)*(x_3-mu_3)^2*(x_4-mu_4)]
        1     3     4     4;  % E[(x_1-mu_1)*(x_3-mu_3)*(x_4-mu_4)^2]
        1     4     4     4;  % E[(x_1-mu_1)*(x_4-mu_4)^3]
        ];
        order =  3; % The truncation order of the moment equations

        % Applying the user-defined closure
        [hoM_closure,flag,moment_closure_rest] = ud_closure(hoM, order, System)

        % The outputs returned by ud_closure are as follow
        flag = [1;0;0;0;1;0;0;1;0;1]
        % Representation of the higher--order moments with flag 1 in terms of lower-order moments
        hoM_closure = [3*C_1_1*C_1_4;
                                   0;
                                   0;
                                   0;
         2*C_1_2*C_2_4 + C_1_4*C_2_2;
                                   0;
                                   0;
         2*C_1_3*C_3_4 + C_1_4*C_3_3;
                                   0;
                       3*C_1_4*C_4_4]
           % The moment closure that should be used for the higher-order moments with flag 0
           moment_closure_rest = 'low-dispersion'
```

To use a user-defined closure for the 2rd-order MM or MCM with 2nd-order outputs for the calculation of the molecule numbers of species in the three-stage gene expression example, the following should be specified:

```
modelName = 'geneExpressionMM'; % The name that will be used for naming the simulation files
method = 'MEC_2_UD_2_a'; % Specifying the modeling approach
System_MM = genSimFile(modelName,modelDefName,method)
```

or

```
modelName = 'geneExpressionMCM'; % The name that will be used for naming the simulation files
method = 'CMEC_2_UD_2_a'; % Specifying the modeling approach
System_MCM = genSimFileIDA(modelName,modelDefName,method)
```

In this case, the user-defined closure should be defined as a function, named `ud_closure.m`, and should be put on the MATLAB path.

Chapter 5

Numerical simulation and sensitivity analysis

The compiled simulation files can be used for numerical simulation and sensitivity analysis for given parameter values.

5.1 Numerical simulation of the system

The numerical simulation can be simply carried out for given parameter values and time vector by using one of the following commands:

- In case either of the MM or the FSP was selected as the modeling approach

```
[status, tout, x, y] = simulate_modelName(t,θ,kappa)
```

- In case the SSE was selected as the modeling approach

```
[status, tout, x, mx, y] = simulate_modelName(t,θ,kappa)
```

- In case the MCM was selected as the modeling approach

```
[status, tout, x, dx, mx, y] = simulate_modelName(t,θ,kappa)
```

The input arguments are:

- `t` is the time vector for which the simulation is to be performed.
- `θ` is the vector of parameter values.
- `kappa` is the vector of constant parameter values (see Section 3.1). This input argument can be left out if there are no constant parameters.

And the output arguments are:

- `status` is a flag indicating whether the numerical simulation was successful or not. This flag is returned by CVODES and IDAS solvers, and can have the following values:

status	CVODES flag
0	CV_SUCCESS
1	CV_TSTOP_RETURN
2	CV_ROOT_RETURN
99	CV_WARNING
-1	CV_TOO_MUCH_WORK
-2	CV_TOO_MUCH_ACC
-3	CV_ERR_FAILURE
-4	CV_CONV_FAILURE
-5	CV_LINIT_FAIL
-6	CV_LSETUP_FAIL
-7	CV_LSOLVE_FAIL
-8	CV_RHSFUNC_FAIL
-9	CV_FIRST_RHSFUNC_ERR
-10	CV_REPTD_RHSFUNC_ERR
-11	CV_UNREC_RHSFUNC_ERR
-12	CV_RTFUNC_FAIL

Simply put, all status values smaller than zero indicate that the integration of the ODE/DAE system failed. Precise interpretation of the CVODES and IDAS flags can be found in the corresponding. documentations [16].

- `tout` is the time vector for which the simulation results are given.
- `x` is the simulation results for the state variables. It is a matrix in which each column is a state variable and each row is a time point.
- `y` is the simulation results for the output variables. It is a matrix in which each column is an output variable and each row is a time point.
- `mx` is the simulation results for the moments of species. It is a matrix in which each column is a species and each row is a time point.
- `dx` is the simulation results for the time-derivative of the state variables. It is a matrix in which each column is a state variable and each row is a time point.

Attention! The meaning of state and output variables (`x` and `y`) is specific to the selected modeling approach (see Section 4.2).

Alternatively, regardless of the modeling approach, the user can call the simulation file with one output argument as below

```
System.sol = simulate_modelName(t,θ,kappa)
```

In this way, the model-specific output arguments above will be added as different fields in `System.sol`. For more information, see Section 5.4.

The above commands will carry out the numerical simulation with the default solver options. The options are documented in the `simulate_modelName.m` file. User-specified solver options can be given as additional input arguments:

```
[···] = simulate_modelName(t, θ, κ, options)
```

Most of the CVODES/IDAS options can be provided. For example, to specify the absolute and relative tolerances and the maximum number of integration steps, the following fields in the `options` structure array can be set:

```
options.cvodes_atol, options.cvodes_rtol, options.cvodes_maxsteps
```

For a complete list of solver options, we refer the user to the documentation in the `simulate_modelName.m` file.

Example: The three-stage gene expression model

Using the MEX-file compiled in Section 4.4, the 2nd-order conditional moment equations for the three-stage gene expression example can be solved for given parameter values and time vector as follows:

```
theta = [0.3;0.3;10;1;4;1;0.015;1;0;0]; % Parameter values
kappa = 1; % Constant parameters (Omega)
t = linspace(0,100,500); % Time vector
[status_MCM,tout_MCM,x_MCM,dx_MCM,mx_MCM,y_MCM] = simulate_geneExpressionMCM(t,theta,kappa);
```

where

- `x_MCM` is a matrix containing the simulation results for the marginal probabilities of `DNA_off` and `DNA_on`, and the 1st and 2nd-order conditional moments of `mRNA` and `Protein` molecule numbers.
- `dx_MCM` is a matrix containing the simulation results for the time-derivative of the marginal probabilities of `DNA_off` and `DNA_on`, and the time-derivative of the 1st and 2nd-order conditional moments of `mRNA` and `Protein` molecule numbers.
- `mx_MCM` is a matrix containing the simulation results for the 1st and 2nd-order overall moments of `DNA_off`, `DNA_on`, `mRNA` and `Protein` molecule numbers.
- `y_MCM` is a matrix containing the simulation results for the 1st and 2nd-order moments of the output variable, i.e. the scaled abundance of `Protein`.

Similarly, to obtain the MM simulation results using the MEX-file compiled in Section 4.4 with specific solver accuracy the following code can be used:

```
theta = [0.3;0.3;10;1;4;1;0.015;1;0;0]; % Parameter values
kappa = 1; % Fixed parameters (Omega)
t = linspace(0,100,500); % Time vector
options.cvodes_atol = 1e-8; % Specifying the absolute tolerance of the numerical solver
options.cvodes_rtol = 1e-8; % Specifying the relative tolerance of the numerical solver
System_MM.sol = simulate_geneExpressionMM(t,theta,kappa,options);
```

- `System_MM.sol.x` is a matrix containing the simulation results for the 1st, 2nd and 3rd-order moments of species, i.e. `DNA_off`, `DNA_on`, `mRNA` and `Protein` concentrations.
- `System_MM.sol.y` is a matrix containing the simulation results for the 1st and 2nd-order moments of the output variable, i.e. the scaled concentration of `Protein`.

5.2 Numerical simulation with arbitrary initial conditions

The initial conditions used in compiling the MEX-files can be rewritten for the numerical simulation. The user can provide the initial conditions for numerical simulation by extending the input argument `kappa`. More specifically, the same command should be used

```
[···] = simulate_modelName(t, θ, κ, options)
```

Here,

$$\kappa = [\mathbf{k}; \mathbf{i}_\mu; \mathbf{i}_C; \mu_0; \mathbf{C}_0]$$

where

- $\mathbf{k} = [k_1; k_2; \cdots]$ is the vector of constant parameter values (see Section 3.1).
- $\mathbf{i}_\mu = [i_{\mu_1}; i_{\mu_2}; \cdots]$ is a vector of the same size as `System.state.mu0` whose elements are either zero or one. Zero indicates that the previously specified value for the corresponding initial condition should be used. One indicates that the new value, provided in κ, for the corresponding initial condition should be used.
- $\mathbf{i}_C = [i_{C_1}; i_{C_2}; \cdots]$ is a vector of the same size as `System.state.C0` whose elements are either zero or one. Zero indicates that the previously specified value for the corresponding initial condition should be used. One indicates that the new value, provided in κ, for the corresponding initial condition should be used.
- $\mu_0 = [\mu_{0_1}; \mu_{0_1}; \cdots]$ is a vector of the same size as `System.state.mu0` including the new initial conditions for the mean of species in the same order as in `System.state.mu0`.
- $\mathbf{C}_0 = [C_{0_1}; C_{0_1}; \cdots]$ is a vector of the same size as `System.state.C0` including the new initial conditions for the (co)variances of species in the same order as in `System.state.C0`.

5.3 Numerical simulation and likelihood evaluation

In order to simulate the system and evaluate the value of a likelihood function for user-provided data and given parameter values, the following command can be used:

```
System.sol = llh_modelName(t,θ,kappa,data,options)
```

The input arguments are:

- `t` is the time vector for which the simulation is to be performed.
- `θ` is the vector of parameter values.
- `kappa` is the vector of constant parameter values (see Section 3.1).
- `data` is a structure array containing the information about the data:
  - `data.Y` contains the values of the measurement data. It is a matrix of the same size as the output variables `y` (see Section 4.2), where each column corresponds to an output variable and each row corresponds to a time point.
  - `data.Sigma_Y` contains the standard deviation of the data. It is a matrix of the same size as `data.Y`.
- `options` (optional) specifies the options of the CVODES solver. We refer the user to the documentation in the `llh_modelName.m` file for a complete list of options.

The output argument `System.sol` includes several fields about the simulation results, some of which are listed below:

- `System.sol.x` is the simulation results for the state variables. It is a matrix in which each column is a state variable and each row is a time point.
- `System.sol.y` is the simulation results for the output variables. It is a matrix in which each column is an output variable and each row is a time point.

- System.sol.llh is the negative log-likelihood value.
- System.sol.sllh is the sensitivity of the negative log-likelihood function with respect to the parameters of interest (see Section 5.4 for more info).
- System.sol.s2llh is the Hessian of the negative log-likelihood function with respect to the parameters of interest.

Example: The three-stage gene expression model

Using the simulation file compiled in Section 4.4 for RRE simulation, the following excerpt of code simulates the system and calculates the value of the negative log-likelihood function.

```
theta = [0.3;0.3;10;1;4;1;0.015;1;0;0]; % Parameter values
kappa = 1; % Fixed parameters (Omega)
t = 1:10; % Time vector
data.Y = [1;5;10;14;18;21;23;25;26;27];
data.Sigma_Y = 0.1*ones(length(t),1);
System_RRE.sol = llh_geneExpressionRRE(t,theta,kappa,data);
```

5.4 Sensitivity analysis

More output arguments can be appended to the commands above to obtain the sensitivities:

```
[status, tout, x, y, sx, sy] = simulate_modelName(t,θ,...)
```

```
[status, tout, x, mx, y, sx, smx, sy] = simulate_modelName(t,θ,...)
```

```
[status, tout, x, dx, mx, y, sx, sdx, smx, sy] = simulate_modelName(t,θ,...)
```

where

- sx is the sensitivity of state variables with respect to parameters. It is a 3-dimensional matrix in which the first dimension corresponds to time points, second dimension corresponds to state variables and third dimension corresponds to parameters.
- sy is the sensitivity of output variables with respect to parameters. It is a 3-dimensional matrix in which the first dimension corresponds to time points, second dimension corresponds to output variables and third dimension corresponds to parameters.
- smx is the sensitivity of the overall moments of species with respect to parameters. It is a 3-dimensional matrix in which the first dimension corresponds to time points, second dimension corresponds to the moments of species and third dimension corresponds to parameters.
- sdx is the sensitivity of state derivatives with respect to parameters. It is a 3-dimensional matrix in which the first dimension corresponds to time points, second dimension corresponds to the state derivatives and the third dimension corresponds to parameters.

Attention! The meaning of state and output variables (x and y) is specific to the selected modeling approach (see Section 4.2).

When the simulation file is called with one output argument as below

```
System.sol = simulate_modelName(t,θ,...)
```

by default the sensitivities are calculated.

Alternatively, the following setting can be used to switch on/off the calculation of the sensitivity equations:

```
options.sensi = 1      or      options.sensi = 0
```

If the sensitivity calculation is switched on, CERENA calculates the sensitivities with respect to all non-constant parameters (see Section 3.1). However, the user can specify the indices of parameters for which the sensitivity equations are to be solved:

```
options.sens_ind = par_ind
```

where `par_ind` is the specified list of parameter indices.

For sensitivity calculation, either forward or adjoint sensitivity equations can be used.

5.4.1 Forward sensitivity analysis

The simulation files generated using `cvodewrap` and `idawrap` by default use forward sensitivity analysis to calculate sensitivities of state and output variables. The sensitivity method can be set to forward sensitivity analysis by the following field in the `options` structure:

```
options.sensi_meth = 1
```

In this case, first the forward sensitivity equations for the state variables are solved, and then the sensitivity of the output variables are calculated based on the state sensitivities.

A list of options specific to the calculation of forward sensitivities can be looked up in the documentation of `simulate_modelName.m`.

Example: The three-stage gene expression model

To solve the forward sensitivity equations for the three-stage gene expression example using IOS simulation (MEX-file compiled in Section 4.4) with arbitrary settings, the following code can be used:

```
theta = [0.3;0.3;10;1;4;1;0.015;1;0;0]; % Parameter values
t = linspace(0,100,500); % Time vector
kappa = 1; % Constant parameters (Omega)
options.sensi = 1; % To enable sensitivity analysis
% Specifying forward sensitivity analysis as the sensitivity calculation method
options.sensi_meth  = 1;
% Indices of the parameters for which the sensitivities are to be calculated.
options.sens_ind = 1:7;
[status_IOS,tout_IOS,x_IOS,mx_IOS,y_IOS,sx_IOS,smx_IOS,sy_IOS] =
                                        simulate_geneExpressionIOS(t,theta,kappa,options);
```

where

- `x_IOS` is a 500×48 matrix containing the simulation results for the IOS states.
- `mx_IOS` is a 500×34 matrix containing the simulation results for the 1st, 2nd and 3rd-order moments of species.
- `y_IOS` is a 500×2 matrix containing the simulation results for the 1st and 2nd-order moments of the output variable, i.e. the scaled concentration of `Protein`.

- `sx_IOS` is a $500 \times 48 \times 7$ matrix containing the simulation results for the sensitivity of IOS states with respect to the first 7 parameters.
- `smx_IOS` is a $500 \times 34 \times 7$ matrix containing the simulation results for the sensitivity of the 1st, 2nd and 3rd-order moments of species with respect to the first 7 parameters.
- `sy_IOS` is a $500 \times 2 \times 7$ matrix containing the simulation results for the sensitivity of output variables, i.e., the mean and the variance of the scaled concentration of `Protein`, with respect to the first 7 parameters.

5.4.2 Adjoint sensitivity analysis

In `cvodewrap` and `llhwrap`, the sensitivity method can be set to adjoint sensitivity analysis by the following field in the `options` structure:

```
options.sensi_meth = 2
```

In this case, *merely* the adjoint sensitivity equations for the output variables are solved. Therefore the output argument `sx` is returned as a zero matrix. When using `cvodewrap`, `sy` will contain the sensitivity of output variables (see Section 4.2) with respect to the parameters. In `llhwrap` `System.sol.sllh` will contain the sensitivity of the negative log-likelihood function with respect to the parameters (see Section 5.3).

A list of options specific to the calculation of adjoint sensitivities can be looked up in the documentation of `simulate_modelName.m` or `llh_modelName.m`.

Example: The three-stage gene expression model

To calculate the adjoint sensitivity equations for the three-stage gene expression example using IOS simulation (MEX-file compiled in Section 4.4), the following is used:

```
theta = [0.3;0.3;10;1;4;1;0.015;1;0;0]; % Parameter values
t = linspace(0,100,500); % Time vector
kappa = 1; % Constant parameters (Omega)
options.sensi = 1; % To enable sensitivity analysis
% Specifying adjoint sensitivity analysis as the sensitivity calculation method
options.sensi_meth  = 2;
% Indices of the parameters for which the sensitivities are to be calculated.
options.sens_ind = 1:7;
[status_IOS,tout_IOS,x_IOS,mx_IOS,y_IOS,sx_IOS,smx_IOS,sy_IOS] =
                                        simulate_geneExpressionIOS(t,theta,kappa,options);
```

In this case, `sx` will be a zero matrix, `smx` will contain the adjoint sensitivities of the moments of species, and `sy` will contain the adjoint sensitivities of the output variables, i.e., the mean and the variance of the scaled concentration of `Protein`.

5.5 MATLAB-based simulation file

In addition, a simulation file entitled `method_modelName_matlab.m` is generated that uses MATLAB `ode15s` solver for the numerical simulation of the system:

- In case either of the MM or the FSP was selected as the modeling approach

```
[tout, x, y] = method_modelName_matlab(t, θ, kappa)
```

- In case either of the SSE or the MCM was selected as the modeling approach

```
[tout, x, mx, y] = method_modelName_matlab(t, θ, kappa)
```

The input arguments are:

- `t` is the time vector for which the simulation is to be performed,
- `θ` is the vector of parameter values.
- `kappa` is the vector of constant parameter values (see Section 3.1). This input argument can be left out if there are no constant parameters.

The output arguments are:

- `tout` is the time vector for which the simulation results are given,
- `x` is the simulation results for the state variables. It is a matrix in which each column is a state variable and each row is a time point.
- `y` is the simulation results for the output variables. It is a matrix in which each column is an output variable and each row is a time point.
- `mx` is the simulation results for the overall moments of species. It is a matrix in which each column is a moment and each row is a time point.

The solver options for `ode15s` can be altered by the user in the `method_modelName_matlab.m` file.

This m-file can be used to perform numerical simulations if the user does not wish to compile MEX-files. However, as far as the computational efficiency is concerned, the usage of this m-file for numerical simulation is not recommended. Unlike the simulation based on MEX-files, the simulation based on m-files does not offer the calculation of sensitivities.

5.6 MATLAB-based FSP simulation

If the state space of the FSP is large, generation of the symbolic representation and compilation of the simulation MEX-files can be time-consuming. Using the command below, the FSP simulation can be performed without generating the symbolic representation of the system. In this case, MATLAB `ode15s` solver is used to solve the system of FSP equations.

```
[x,Mx,My,cMx] = simulateFSP_matlab(modelName,t,θ,kappa,System_MCM,options)
```

or

```
System = simulateFSP_matlab(modelName,t,θ,kappa,System_MCM,options)
```

The input arguments are:

- `modelName` is the name of the `modelDef.m` file.
- `t` is the time vector for which the simulation is to be performed.
- `θ` is the vector of parameter values.
- `kappa` is the vector of constant parameter values (see Section 3.1).
- `System_MCM` (optional) is the `System` structure obtained from the MCM simulation. This input argument is required if the conditional moments based on the FSP simulation are to be calculated.
- `options` (optional) specifies the following:

  - options.ode15s.reltol sets the relative tolerance of ode15s.
  - options.ode15s.abstol sets the absolute tolerance of ode15s.
  - options.moment_order specifies which moments of the species should be calculated.
  - options.moment_order_output specifies which moments of the output variables should be calculated.

The output arguments are:

- x is the simulation results for the FSP states.
- Mx is the simulation results for the moments of the species up to order options.moment_order.
- My is the simulation results for the moments of the output variables up to order options.moment_order_output.
- cMx is the simulation results for the marginal probabilities and the conditional moments of the species in the same order as in System_MCM.

Chapter 6

SSA simulation

6.1 Description

In addition to all the modeling approaches that yield a system of differential equations (see Section 4.2), CERENA offers SSA simulations [3]. Using the following command, CERENA generates statistically representative realizations of the state of the biochemical reaction network:

```
System = simulateSSA_matlab(modelName,t,θ,kappa,Nssa,options);
```

where

- `modelName` is the name of the `modelDef.m` file.
- `t` is the vector of time points at which the SSA results are to be stored.
- `θ` is the parameter values used for the SSA simulation.
- `kappa` is the vector of constant parameter values (see Section 3.1).
- `Nssa` (optional) is the number of SSA realizations to be simulated. The default value is set to 10.
- `options` (optional) specifies the options for SSA simulation, as explained below.

The output arguments are as follow:

- `System.sol.x` is a 3-dimensional matrix which contains the state of the system, i.e. the count of species, at the time points specified by `t` vector. The first dimension corresponds to time points, the second dimension corresponds to species, and the third dimension corresponds to individual realizations.
- `System.sol.y` is a 3-dimensional matrix which contains the state of output variables at the time points specified by `t` vector. The first dimension corresponds to time points, the second dimension corresponds to output variables, and the third dimension corresponds to individual realizations.
- `System.sol.mean_x` is a 2-dimensional matrix which contains the mean of concentrations/counts of species at the time points specified by `t` vector. The first dimension corresponds to time points, and the second dimension corresponds to species. The means are calculated by Monte-Carlo integration over individual realizations (see Section 1.3.1).
- `System.sol.mean_y` is a 2-dimensional matrix which contains the mean concentrations/counts of output variables at the time points specified by `t` vector. The first dimension corresponds to time points, and the second dimension corresponds to output variables. The means are calculated by Monte-Carlo integration over individual realizations (see Section 1.3.1).

- `System.sol.var_x` is a 2-dimensional matrix which contains the variance of concentrations/counts of species at the time points specified by `t` vector. The first dimension corresponds to time points, and the second dimension corresponds to species. The variances are calculated by Monte-Carlo integration over individual realizations (see Section 1.3.1).
- `System.sol.var_y` is a 2-dimensional matrix which contains the variance of concentrations/counts of output variables at the time points specified by `t` vector. The first dimension corresponds to time points, and the second dimension corresponds to output variables. The variances are calculated by Monte-Carlo integration over individual realizations (see Section 1.3.1).

6.1.1 Constant propensities

If the propensities do not explicitly depend on time, i.e. $a_j = a_j(\mathbf{x})$, the following option should be provided when calling `simulateSSA_matlab`:

```
options.mode = 'constant'
```

In this case, which is the default mode for SSA simulations, a next-reaction method [3] is performed to generate the SSA trajectories.

6.1.2 Time-dependent propensities

If the propensities are time-dependent, i.e. $a_j = a_j(\mathbf{x}, t)$, the following option should be provided when calling `simulateSSA_matlab`:

```
options.mode = 'time-dependent'
```

In this case, CERENA uses a variation of the next-reaction method for time-dependent propensities [4] to simulate the trajectories of the system.

Other options

To specify whether the counts or the concentrations of species should be simulated, the following option can be used:

`options.scale = 'absolute'  (default)` *or* `options.scale = 'concentration'`

6.2 Example: The three-stage gene expression model

The following code can be used to generate 10000 trajectories for the three-stage gene expression example with given parameter values and time vector:

```
modelDefName = 'modelDef_geneExpression';
theta = [0.3;0.3;10;1;4;1;0.015;1;0;0];
kappa = 1;
t = linspace(0,100,500);
Nssa = 10000;
options.mode = 'constant';
options.scale = 'absolute';
System_SSA = simulateSSA_matlab(modelDefName,t,theta,kappa,Nssa,options);
```

With the following output arguments:

- `System_SSA.sol.x` is a $500 \times 4 \times 10000$ matrix which contains the counts of `DNA_off`, `DNA_on`, `mRNA` and `Protein` at the time points specified by `t` vector.
- `System_SSA.sol.y` is a $500 \times 1 \times 10000$ matrix which contains the value of the scaled `Protein` count at the time points specified by `t` vector.
- `System_SSA.sol.mean_x` is a 500×4 matrix which contains the mean of the counts of `DNA_off`, `DNA_on`, `mRNA` and `Protein` at the time points specified by `t` vector.
- `System_SSA.sol.mean_y` is a 500×1 matrix which contains the mean of the scaled `Protein` count at the time points specified by `t` vector.
- `System_SSA.sol.var_x` is a 500×4 matrix which contains the variance of the counts of `DNA_off`, `DNA_on`, `mRNA` and `Protein` at the time points specified by `t` vector.
- `System_SSA.sol.var_y` is a 500×1 matrix which contains the variance of the scaled `Protein` count at the time points specified by `t` vector.

Chapter 7

Post-processing and visualization of simulation results

The results of the numerical simulation can be visualized using CERENA plotting routines. For each of the modeling approaches, a specific plotting routine can be called as follows.

7.1 Visualization of trajectories

For the visualization of the simulation results, CERENA provides a variety of simple line plots for the different modeling approaches. For the FSP simulations, routines for the visualization of the probability distributions are implemented.

7.1.1 Visualization of MM simulation results

In MM simulations, the following command can be used to provide plots of the simulation results:

```
plotMM(System ,options)
```

where

- `System` is the `System` structure obtained from the MM simulation.
- `options` contains specifications for plotting. This input argument is optional and can be left out.

If no options are specified, `plotMM` will generate two figures illustrating the time courses of the mean of species and output variables, including the 1-σ intervals, $[m - \sigma, m + \sigma]$. Here, σ denotes the standard deviation. To plot the higher-order moments of species and output variables, the following options can be set and passed to `plotMM`:

```
options.plot_xo = 1, options.state_order = xo
```

```
options.plot_yo = 1, options.output_order = yo
```

where

- `xo` specifies the moment order of interest for species.

- `yo` specifies the moment order of interest for output variables.

In this case, `xo` figures will be generated displaying the time courses of the first `xo` moments of species. Similarly, `yo` figures will be generated displaying the time courses of the first `yo` moments of output variables.

7.1.2 Visualization of SSE simulation results

In SSE simulations, the following command can be used to provide plots of the simulation results:

```
plotSSE(System ,options)
```

where

- `System` is the `System` structure obtained from the SSE simulation.
- `options` contains specifications for plotting. This input argument is optional and can be left out.

If no options are specified, `plotSSE` will generate a figure illustrating the time courses of the SSE state variables. In addition, it generates two figurea where the time courses of the mean of the species as well as the output variables, including the 1-σ intervals, $[m - \sigma, m + \sigma]$, are plotted. To plot the higher-order moments of species and output variables, the following options can be set and passed to `plotSSE`:

```
options.plot_xo = 1, options.plot_yo = 1
```

In this case, the time courses of the 2nd-order moments (for LNA, EMRE and IOS) and the 3rd-order moments (for IOS) of species and output variables are plotted.

7.1.3 Visualization of MCM simulation results

In MCM simulations, the following command can be used to provide plots of the simulation results:

```
plotMCM(System ,options)
```

where

- `System` is the `System` structure obtained from the MCM simulation.
- `options` contains specifications for plotting. This input argument is optional and can be left out.

If no options are specified, `plotMCM` will generate a figure illustrating the time courses of the marginal probabilities of the `'stochastic'` species and the conditional mean of `'moment'` species including the 1-σ intervals, $[m - \sigma, m + \sigma]$. In addition, a figure displaying the time courses of the mean of output variables including the 1-σ intervals, $[m - \sigma, m + \sigma]$, is generated. To plot the higher-order moments of species and output variables, the following options can be set and passed to `plotMCM`:

```
options.plot_xo = 1, options.state_order = xo
```

```
options.plot_yo = 1, options.output_order = yo
```

where

- `xo` is the moment order of interest for species.
- `yo` is the moment order of interest for output variables.

In this case, `xo` figures will be generated displaying the time courses of the first `xo` conditional moments of `'moment'` species. Similarly, `xo` figures will be generated displaying the time courses of the first `xo` overall moments of species. In addition, `yo` figures will be generated displaying the time courses of the first `yo` moments of output variables.

7.1.4 Visualization of FSP simulation results

In FSP simulations, the following command can be used to provide plots of the simulation results:

```
plotFSP_CERENA(System ,options)
```

where

- `System` is the `System` structure obtained from the FSP simulation.
- `options` contains specifications for plotting. This input argument is optional and can be left out.

If no options are specified, `plotFSP_CERENA` will generate a figure visualizing the probability distribution over the FSP states. In addition, the time courses of the mean of the species and the output variables, including the 1-σ intervals $[m-\sigma, m+\sigma]$, are plotted. To plot the higher-order moments of the species and the output variables, the following options can be set and passed to `plotFSP_CERENA`:

```
options.plot_xo = 1, options.state_order = xo
```

```
options.plot_yo = 1, options.output_order = yo
```

In this case, `xo` figures will be generated displaying the time courses of the first `xo` moments of species. Similarly, `yo` figures will be generated displaying the time courses of the first `yo` moments of output variables.

7.1.5 Visualization of SSA simulation results

The results of SSA simulation can be visualized using the following command:

```
plotSSA(System ,options)
```

where

- `System` is the `System` structure obtained from the SSA simulation.
- `options` contains specifications for plotting. This input argument is optional and can be left out.

If no options are provided, `plotSSA` generates three figures: The first figure displays the counts of species and output variables in 10 randomly chosen realizations. To plot more realizations, the following option can be provided:

```
options.n_realization = nr
```

In this case, `nr` randomly chosen realizations are visualized. The second and third figure show the time courses of the mean of species and output variables including the 1-σ intervals $[m-\sigma, m+\sigma]$. To plot the higher-order moments of species and output variables the following options can be set and passed to `plotSSA`:

```
options.plot_xo = 1, options.state_order = xo
```

```
options.plot_yo = 1, options.output_order = yo
```

In this case, `xo` figures will be generated displaying the time courses of the first `xo` moments of species. Similarly, `yo` figures will be generated displaying the time courses of the first `yo` moments of output variables.

7.1.6 Visualization of several simulation results

The visualize and compare the results of several simulations, the following command can be used:

```
plotCompare(SystemAll)
```

where `SystemAll` is a structure array containing the Systems to be visualized, e.g.,

```
SystemAll{1} = System_MM; SystemAll{2} = System_SSA; ···
```

`plotCompare` plots the time courses of the mean and the variance of species and the mean and the variance of the output variables for all Systems included n `SystemAll`.

7.1.7 General options

In addition to the method-specific options, the user can specify general plotting options as below:

- `options.lc` specifies the line color for plotting.
- `options.lw` specifies the line width.
- `options.fs` specifies the font size.
- `options.save` specifies that the figures should be saved if set to 1.
- `options.save_path` specifies the path under which the figures should be saved.

7.2 Visualization of correlation and partial correlation

In addition to directly plotting the simulation results, CERENA offers the calculation and visualization of the correlation and partial correction maps for further investigations/interpretations of the simulation results.

7.2.1 Correlation map

The following command can be used to obtain the correlation map:

```
[corrMat,corrMatAll,covMat] = corrmat(System ,System.sol.x,options);
```

where

- `options` contains specifications for plotting. This input argument is optional and can be left out.
- `corrMat` is a matrix consisting of the maximum correlation coefficients across all time points.
- `corrMatAll` is a 3-dimensional matrix of all correlation coefficients across all time points. The first two dimensions correspond to variables and the third dimension corresponds to time points.
- `covMat` is a covariance matrix corresponding to maximum correlation coefficients across all time points.

If `options.visualization = 'on'` (default), a correlation map consisting of the maximum correlation coefficients across all time points is plotted. Also, a movie of the correlation map over time is generated and entitled `options.movieName`.

7.2.2 Partial correlation map

The following command can be used to obtain the partial correlation map:

```
[pcorrMat,pcorrMatAll] = pcorrmat(System ,System.sol.x,covMat,corrMatAll,options)
```

where

- `covMat` is a 3-dimensional matrix which contains the covariance matrix over all time points. The first two dimensions correspond to variables and the third dimension corresponds to time points.
- `corrMatAll` is given by `corrmat` and is a 3-dimensional matrix of all the correlation coefficients across all time points. The first two dimensions correspond to variables and the third dimension corresponds to time points.
- `options` contains specifications for plotting. This input argument is optional and can be left out.
- `pCorrMat` is a matrix consisting of the maximum partial correlation coefficients across all time points.
- `pCorrMatAll` is a 3-dimensional matrix of all the partial correlation coefficients across all time points. The first two dimensions correspond to variables and the third dimension corresponds to time points.

If `options.visualization = 'on'` (default), a partial correlation map consisting of the maximum partial correlation coefficients across all time points is plotted. Also, a movie of the partial correlation map over time is generated and entitled `options.movieName`.

Attention! If necessary, a shrinkage of the covariance matrix is automatically performed to yield a positive semi-definite covariance matrix to be used in the calculation of partial correlation coefficients [19, 20].

7.3 Example: The three-stage gene expression model

The simulation results obtained by MM simulation (see Section 5.1) are plotted as below, and shown in Figure 7.1:

```
plotMM(System_MM)
```

To plot the 2nd and 3rd-order moments of the species (Figure 7.2), the following options are set:

```
options.plot_xo = 1;
options.state_order = 3;
plotMM(System_MM,options)
```

Similarly, the states of the IOS simulation (see Section 5.4.1), together with the moments of species and the output moments, can be visualized as below (see Figure 7.3 and Figure 7.4):

```
plotSSE(System_IOS)
```

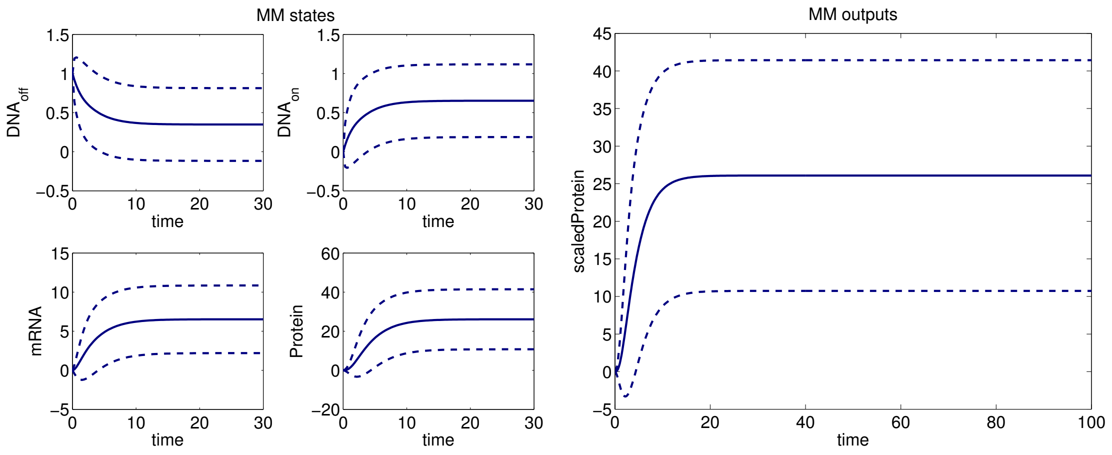

(a) (b)

Figure 7.1: **Visualization of the MM simulation results for the three-stage gene expression example.** (a) Mean of species including the 1-σ intervals. (b) Mean of output variables including the 1-σ intervals.

The results of MM, MCM, and IOS simulations are plotted together as below, and the resulting plots are shown in Figure 7.5:

```
SystemAll{1} = System_MM;
SystemAll{2} = System_MCM;
SystemAll{3} = System_IOS;
plotCompare(SystemAll)
```

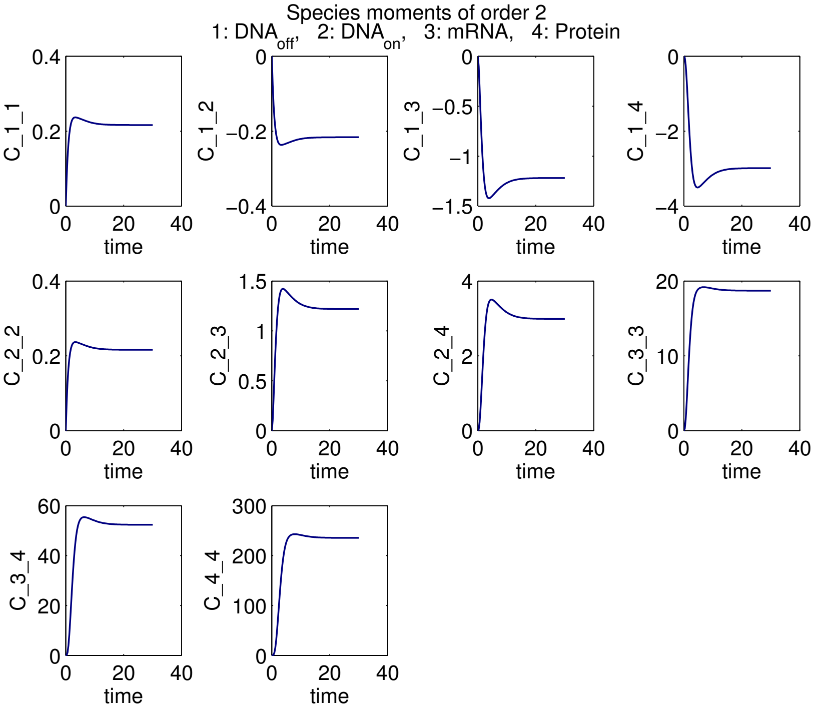

(a)

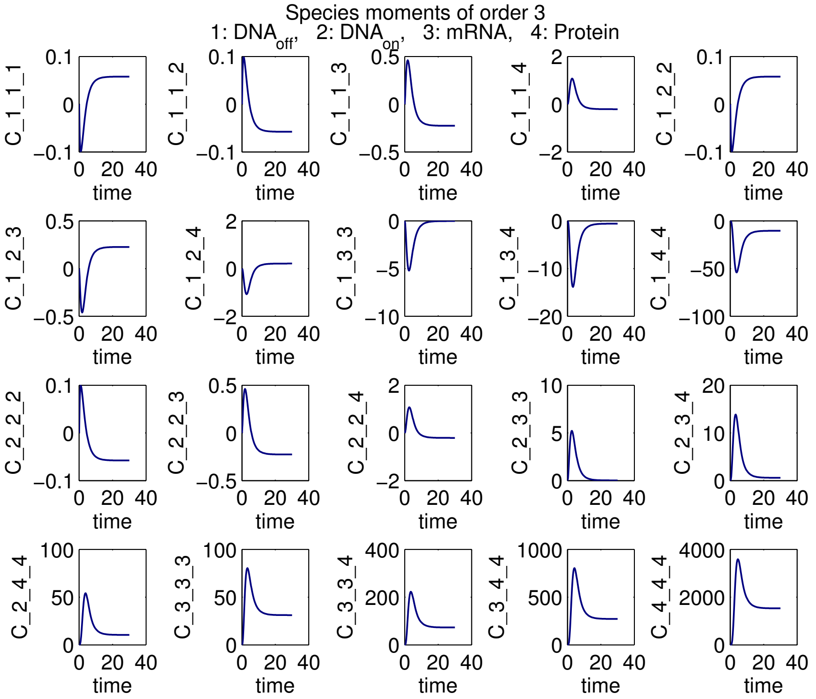

(b)

Figure 7.2: **Visualization of higher-order moments in the three-stage gene expression example using MM simulation.** (a) Second-order moments of species. (b) Third-order moments of species.

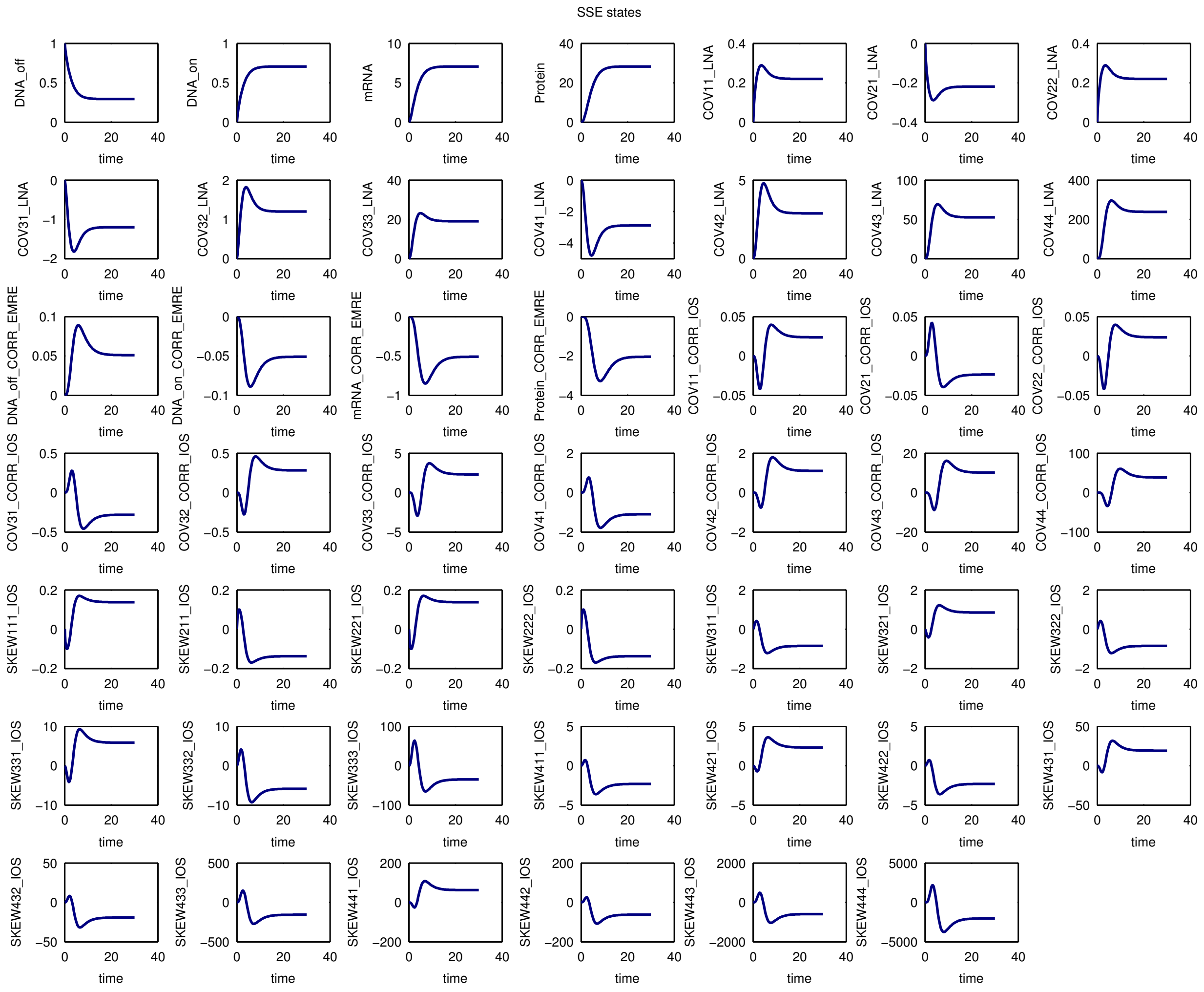

Figure 7.3: **Visualization of the SSE states for the three-stage gene expression example.**

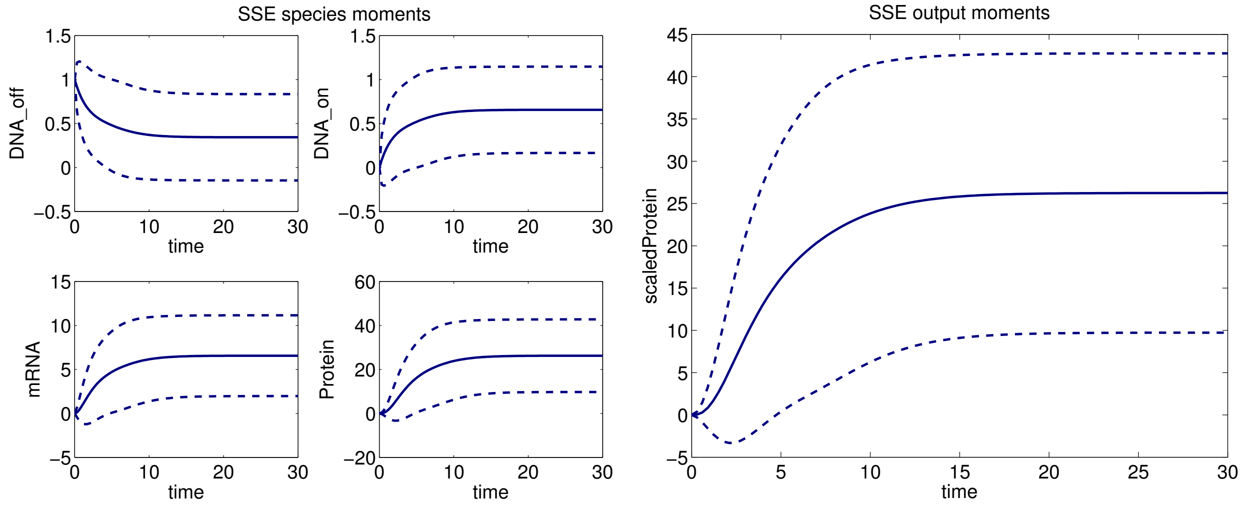

(a)

(b)

Figure 7.4: **Visualization of the moments of species and the output variables in the three-stage gene expression example obtained by the SSE simulation.** (a) Mean of species including the 1-σ intervals. (b) Mean of output variables including the 1-σ intervals.

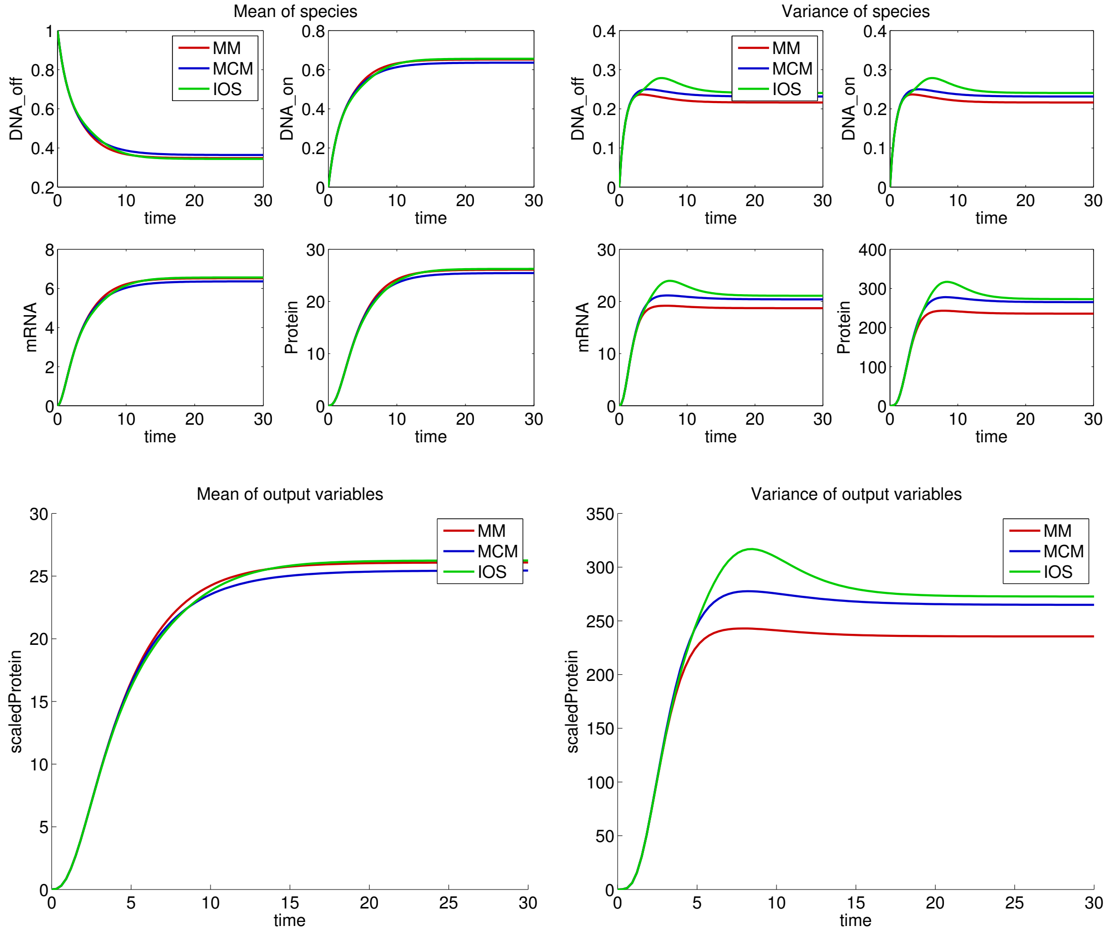

Figure 7.5: **Mean and variance of species and output variables in the three-stage gene expression example obtained by the MM, the MCM and the IOS simulations.**

Chapter 8

Further examples

8.1 JAK-STAT signaling pathway

The second example studied using CERENA is a model of the JAK-STAT signaling pathway introduced by [21]. The model, sketched in Figure 8.1, describes the signaling cascade of STAT protein. Upon activation, Epo receptor triggers the phosphorylation of cytoplasmic STAT. Dimerization and translocation of phosphorylated STAT into the nucleus, followed by a delayed export of STAT from the nucleus complete the loop. The time-dependent concentration of phosphorylated Epo receptor, [pEpoR], functions as an input to the system.

The JAK-STAT signaling pathway is an interesting application example as it (i) includes two compartments, namely cytoplasm and nucleus, and (ii) involves a time-dependent propensity. Given the parameter values and initial conditions in Table A, this pathway is simulated using several modeling approaches, including SSA.

The SSA simulation results, are plotted using the command below, and are shown in Figures 8.2 and 8.3:

```
options.fs = 10;
plotSSA(System_SSA,options)
```

The model definition and simulation files for this system can be found in `CERENA/examples/JakStat`.

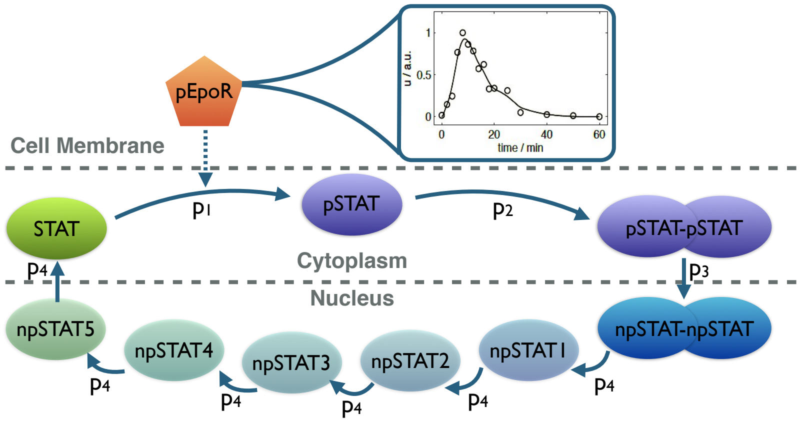

Figure 8.1: Schematic of the simplified JAK-STAT signaling pathway.

Table A: Parameter values and initial conditions of the three-stage gene expression example.

p_1	3.9364
p_2	10
p_3	0.1125
p_4	0.9829
$X_{c,\text{STAT}}(0)$	1
$X_{c,\text{pSTAT}}(0)$	0
$X_{c,\text{pSTAT-pSTAT}}(0)$	0
$X_{c,\text{npSTAT-npSTAT}}(0)$	0
$X_{c,\text{npSTAT1}}(0)$	0
$X_{c,\text{npSTAT2}}(0)$	0
$X_{c,\text{npSTAT3}}(0)$	0
$X_{c,\text{npSTAT4}}(0)$	0
$X_{c,\text{npSTAT5}}(0)$	0

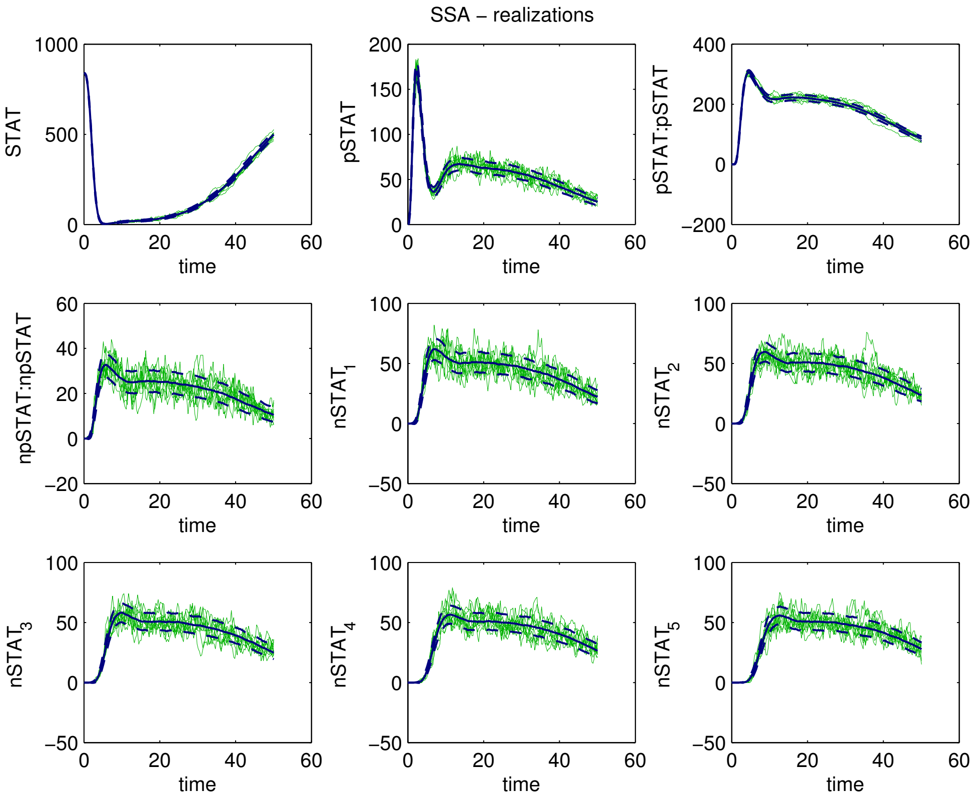

Figure 8.2: **SSA simulation results for the simplified JAK-STAT pathway.** Individual realizations (green) and the mean of the counts of species including 1-σ intervals (blue) are shown.

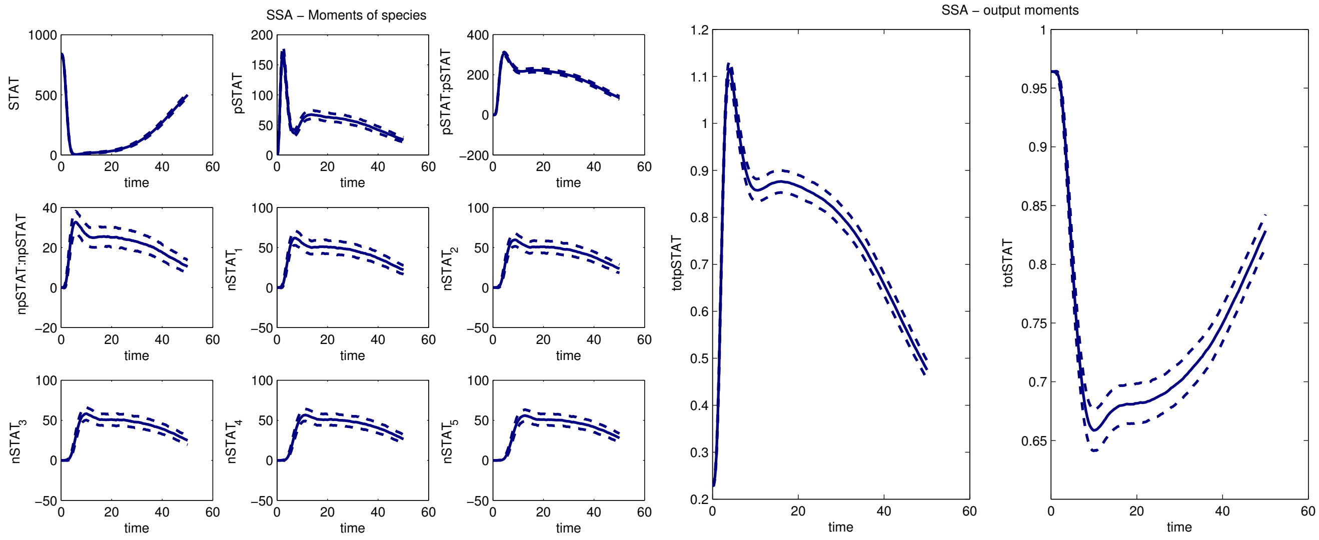

Figure 8.3: **The counts of species and the concentration of the observable, i.e. total concentration of STAT, obtained by SSA simulation.** The means, including 1-σ intervals, are shown.

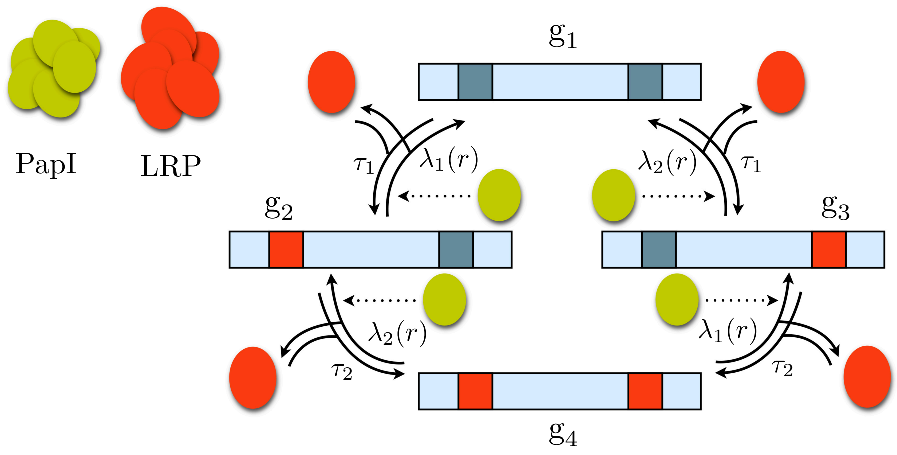

Figure 8.4: **Schematic of the PapI regulation model.** Arrows represent the binding and unbinding of LRP to/from the operon. Dotted arrows indicate the influence of PapI on the reaction rates.

8.2 PapI regulation model

This model, sketched in Figure 8.4, describes the regulation of Pap pili formation on the surface of *E. coli* [22]. This system includes a a *pap* operon and regulatory proteins LRP and PapI. Binding of LRP to either or both of the binding sites results in four configurations of the *pap* operon. Only if the operon is in state g_2, pili can be produced. PapI sets up a a positive feedback loop for the production of pili by reducing the unbinding rate of LRP from the operon.

This process is an interesting application example as it involves non-polynomial propensity functions. The model definition and simulation files for this system can be found in `CERENA/examples/PapI`.

8.3 A gene cascade network

A gene cascade network, described in [13], is implemented in CERENA. The model definition and simulation files can be looked up in `CERENA/examples/geneCascade`.

Bibliography

[1] Shahrezaei V, Swain PS. Analytical distributions for stochastic gene expression. Proc Natl Acad Sci USA. 2008 Nov;105(45):17256–17261.

[2] Norris JR. Continuous-time Markov chains I. In: Markov Chains. Cambridge University Press; 1997. p. 60–107. Cambridge Books Online.

[3] Gillespie DT. Exact stochastic simulation of coupled chemical reactions. J Phys Chem. 1977 Dec;81(25):2340–2361.

[4] Anderson DF. A modified next reaction method for simulating chemical systems with time dependent propensities and delays. J Chem Phys. 2007 Dec;127(214107).

[5] Munsky B, Khammash M. The finite state projection algorithm for the solution of the chemical master equation. J Chem Phys. 2006 Jan;124(4):044104.

[6] van Kampen NG. Stochastic processes in physics and chemistry. 3rd ed. Amsterdam: North-Holland; 2007.

[7] Grima R. Linear-noise approximation and the chemical master equation agree up to second-order moments for a class of chemical systems. Phys Rev E. 2015 Oct;92:042124.

[8] Grima R. An effective rate equation approach to reaction kinetics in small volumes: Theory and application to biochemical reactions in nonequilibrium steady-state conditions. J Chem Phys. 2010 July;133(035101).

[9] Thomas P, Matuschek H, Grima R. How reliable is the linear noise approximation of gene regulatory networks? BMC Genomics. 2013 Oct;14(Suppl 4)(S5).

[10] Ramaswamy R, González-Segredo N, Sbalzarini I, Grima R. Discreteness-induced concentration inversion in mesoscopic chemical systems. Nat Comm. 2012 Apr;3(779).

[11] Engblom S. Computing the moments of high dimensional solutions of the master equation. Appl Math Comp. 2006;180:498–515.

[12] Lee CH, Kim KH, Kim P. A moment closure method for stochastic reaction networks. J Chem Phys. 2009 Apr;130(13):134107.

[13] Singh A, Hespanha JP. Approximate moment dynamics for chemically reacting systems. IEEE Trans Autom Control. 2011 Feb;56(2):414–418.

[14] Hasenauer J, Wolf V, Kazeroonian A, Theis FJ. Method of conditional moments (MCM) for the chemical master equation. J Math Biol. 2014 Aug;69(3):687–735.

[15] Raue A, Schilling M, Bachmann J, Matteson A, Schelke M, Kaschek D, et al. Lessons learned from quantitative dynamical modeling in systems biology. PLoS ONE. 2013 Sept;8(9):e74335.

[16] Hindmarsh AC, Brown PN, Grant KE, Lee SL, Serban R, Shumaker DE, et al. SUNDIALS: Suite of Nonlinear and Differential/Algebraic Equation Solvers. ACM T Math Software. 2005 Sept;31(3):363–396.

[17] Keating SM, Bornstein BJ, Finney A, Hucka M. SBMLToolbox: an SBML toolbox for MATLAB users. Bioinformatics. 2006;22(10):1275–1277. Available from: http://bioinformatics.oxfordjournals.org/content/22/10/1275.abstract.

[18] Raue A, Steiert B, Schelker M, Kreutz C, Maiwald T, Hass H, et al. Data2Dynamics: a modeling environment tailored to parameter estimation in dynamical systems. Bioinformatics. 2015 Jul.

[19] Schafer J, Strimmer K. A shrinkage approach to large-scale covariance matrix estimation and implications for functional genomics. Stat Appl Genet Mol Biol. 2005;4:Article32.

[20] Opgen-Rhein R, Strimmer K. Accurate ranking of differentially expressed genes by a distribution-free shrinkage approach. Stat Appl Genet Mol Biol. 2007;6:Article9.

[21] Raue A, Kreutz C, Maiwald T, Bachmann J, Schilling M, Klingmüller U, et al. Structural and practical identifiability analysis of partially observed dynamical models by exploiting the profile likelihood. Bioinf. 2009 May;25(25):1923–1929.

[22] Kazeroonian A, Theis FJ, Hasenauer J. Modeling of stochastic biological processes with non-polynomial propensities using non-central conditional moment equation. In: Proc. of the 19th IFAC World Congress. vol. 19. Cape Town, South Africa; 2014. p. 1729–1735.

Acronyms

CERENA **C**h**E**mical **RE**action **N**etwork **A**nalyzer: A toolbox for the simulation and analysis of chemical reaction networks using approximations of the Chemical Master Equation solution statistics. 5

CME Chemical Master Equation. 7–10

DAE Differential Algebraic Equation. 11, 12, 23, 25, 27, 28, 30, 32

EMRE Effective Mesoscopic Rate Equation. 9, 25, 44

FSP Finite State Projection. 2, 8, 12, 17, 18, 23, 25, 27, 28, 31, 37–39, 43, 45

IOS Inverse Omega Square. 9, 25, 29, 36, 37, 44, 47, 48, 52

LNA Linear Noise Approximation. 9, 25, 44

MCM Method of Conditional Moments. 2, 10, 12, 17, 18, 23, 25–27, 30, 31, 38, 44, 48, 52

MM Method of Moments. 2, 9, 12, 23, 25, 26, 29, 31, 33, 37, 43, 47–49, 52

ODE Ordinary Differential Equation. 7–9, 12, 13, 23–26, 28, 29, 32

RRE Reaction Rate Equations. 8, 9, 12, 23, 25, 35

SSA Stochastic Simulation Algorithms. 2, 7, 40, 41, 45, 53, 55

SSE System Size Expansion. 2, 9, 12, 23, 25, 31, 38, 44, 50, 51

Glossary

`System` is a MATLAB structure array containing the reaction network specifications. 16, 20, 22–24, 32, 34, 35, 37, 38, 40, 41, 43–47, 59

`completeSystem` is a function for constructing the `System` structure. 20, 24

`cvodewrap` is the wrapper for CVODES package. 24, 28, 29, 36, 37, 59

`genSimFileIDA` is a function for generating the simulation MEX-files using `idawrap`. 23, 25, 30

`genSimFileLLH` is a function for generating the simulation MEX-files using `llhwrap`. 23, 25, 30

`genSimFile` is a function for generating the simulation MEX-files using `cvodewrap`. 23–25

`genmexp` is a function for the derivation of the governing equations and the generation of the symbolic representation of the system. 24, 28

`idawrap` is the wrapper for the IDAS package. 25, 28, 29, 36, 59

`llhwrap` is the wrapper for CVODES package which also enables the evaluation of the negative log-likelihood function. 24, 28, 29, 37, 59

`method` is the selected modeling approach. 23–29

`modelDef.m` is the model definition file. 16, 20, 22, 23, 25–28, 38, 40

`modelDefName` is the name of the `modelDef.m` file. 23, 24, 28

`modelName` is the name of the reaction network. This name will be used in naming the simulation files. 23–25, 28, 29, 38, 40
